# Novel Sulfonamide-Based Analogs of Metformin Exert Promising Anti-Coagulant Effects without Compromising Glucose-Lowering Activity

**DOI:** 10.3390/ph13100323

**Published:** 2020-10-21

**Authors:** Magdalena Markowicz-Piasecka, Adrianna Sadkowska, Joanna Sikora, Marlena Broncel, Kristiina M. Huttunen

**Affiliations:** 1 Laboratory of Bioanalysis, Department of Pharmaceutical Chemistry, Drug Analysis and Radiopharmacy, Medical University of Lodz, ul. Muszyńskiego1, 90-151 Lodz, Poland; joanna.sikora@umed.lodz.pl; 2 Students Research Group, Laboratory of Bioanalysis, Department of Pharmaceutical Chemistry, Drug Analysis and Radiopharmacy, Medical University of Lodz, ul. Muszyńskiego 1, 90-151 Lodz, Poland; adrianna.sadkowska@stud.umed.lodz.pl; 3 Department of Internal Diseases and Clinical Pharmacology, Medical University of Lodz, Kniaziewicza 1/5, 91-347 Lodz, Poland; marlena.broncel@umed.lodz.pl; 4 School of Pharmacy, Faculty of Health Sciences, University of Eastern Finland, Yliopistonranta 1C, POB 1627, 70211 Kuopio, Finland; kristiina.huttunen@uef.fi

**Keywords:** hemostasis, coagulation, biguanide, metformin, endothelium

## Abstract

Metformin, one of the most frequently prescribed oral anti-diabetic drugs, is characterized by multidirectional activity, including lipid lowering, cardio-protective and anti-inflammatory properties. This study presents synthesis and stability studies of 10 novel sulfonamide-based derivatives of metformin with alkyl substituents in the aromatic ring. The potential of the synthesized compounds as glucose-lowering agents and their effects on selected parameters of plasma and vascular hemostasis were examined. Compounds with two or three methyl groups in the aromatic ring (**6**, **7**, **9**, **10**) significantly increased glucose uptake in human umbilical vein endothelial cells (HUVECs), e.g., 15.8 µmol/L for comp. **6** at 0.3 µmol/mL versus 11.4 ± 0.7 µmol/L for control. Basic coagulation studies showed that all examined compounds inhibit intrinsic coagulation pathway and the process of fibrin polymerization stronger than the parent drug, metformin, which give evidence of their greater anti-coagulant properties. Importantly, synthesized compounds decrease the activity of factor X, a first member of common coagulation pathway, while metformin does not affect coagulation factor X (FX) activity. A multiparametric clot formation and lysis test (CL-test) revealed that the examined compounds significantly prolong the onset of clot formation; however, they do not affect the overall potential of clot formation and fibrinolysis. Erythrotoxicity studies confirmed that none of the synthesized compounds exert an adverse effect on erythrocyte integrity, do not contribute to the massive hemolysis and do not interact strongly with the erythrocyte membrane. In summary, chemical modification of metformin scaffold into benzenesulfonamides containing alkyl substituents leads to the formation of potential dual-action agents with comparable glucose-lowering properties and stronger anti-coagulant activity than the parent drug, metformin.

## 1. Introduction

In the previous decades, drugs were largely discovered with the use of the “one molecule–one target–one disease” strategy. It allowed to discover many successful drugs, that are selective for a single target known as “magic bullets.” These target-centric drugs are characterized with high selectivity and potency against their specific targets, and undoubtedly, this strategy will continue to be a primary way of drug development. At the same time, several well-known drugs have appeared to affect more than one molecular target, which proved that their efficacy stems from this multi-target behavior. One such example is imatinib, which was originally developed as a selective inhibitor of the BCR-Abl fusion protein, but was also shown to inhibit the non-oncogenic C-Abl kinase [1]. Recently, a growing interest in multi-target drug discovery (MTDD) approach has been observed. The scientific community finds MTDD a superior strategy which contributes to therapeutic effect and side effect profile compared to the modulation of a single target [2]. The use of multi-target drugs, or polypharmacology, acknowledges an agent to simultaneously affect multiple targets or processes; either by a single compound or a combination of several molecules binding to different targets associated with the disease [3].

Type 2 diabetes mellitus (T2DM) is a multifactorial metabolic disease that occurs either when the pancreas does not produce enough insulin or when the body cannot effectively use the insulin it produces. Uncontrolled diabetes is characterized by increased levels of blood glucose (hyperglycemia), which contributes to macrovascular and microvascular complications, finally leading to disabilities and reduction in life expectancy. Numerous studies have shown that diabetes is associated with thrombosis and the activation of blood coagulation [4,5,6]. In addition, T2DM is characterized by an impaired balance between the processes of coagulation and fibrinolysis. For instance, elevated activity of coagulation factors including fibrinogen, von Willebrand factor and coagulation factor VII (FVII) have been associated with diabetes [7,8]. Metabolic disorders and hyperglycemia occurring in the course of diabetes significantly affect the morphology, function and activation of platelets. The platelets are characterized by hyperreactivity, increased adhesiveness, exaggerated aggregation and changed metabolism [8]. Diabetic state is also associated with quantitative and qualitative changes in coagulation proteins that increase fibrinolysis resistance, which in turn increases the risk of thrombosis. Diabetic patients have dense fibrin networks with densely packed thin fibers, which hinders the penetration of fibrinolytic enzymes into the clot and, consequently, increases lysis time [9]. Furthermore, vascular endothelial dysfunction is an initiating factor contributing to the pathogenesis and clinical expression of atherosclerosis and has been clinically linked to T2DM [10,11]. Therefore, it has been fully justified to search for novel compounds with both anti-diabetic and anti-coagulation properties.

Sulfonamides are one of the most frequently appearing structural backbones in synthetic drugs [3]. Since the development of Prontosil, an antibacterial agent, in 1935, thousands of sulfonamide derivatives have been synthesized. The main reason for the development of new sulfonamides is the constantly growing number of resistant bacteria strains. Apart from antibacterial properties sulfonamides were also found to possess a broad spectrum of pharmacological activity, including carbonic anhydrase inhibition (e.g., acetazolamid), anti-viral (amprenavir), diuretic (furosemide) or anti-inflammatory properties (celecoxib, nimesulid) [12]. An important group of drugs being sulfonylurea derivatives (e.g., glimepiride) that has insulin secretion stimulating effects cannot be overlooked when discussing pharmacodynamic properties of sulfonamides [13]. However, the sulfonamide moiety has also been incorporated to known biologically relevant backbones to generate novel biological activity [3]. For instance, new anti-AD (Alzheimer’s disease) agents with inhibitory properties towards butyrylcholinestarase (BChE) have been synthesized by incorporation of sulfonamide moiety to earlier hit compounds [14]. These results suggest that sulfonamide-based compounds exert multi-directional biological activity. In addition, this structural versatility of sulfa-drugs and well-established available information make them promising candidates for developing more effective alternatives to the currently-approved drugs.

Metformin, a synthetic biguanide, is a first-line treatment of T2DM patients. The glucose-lowering mechanism of metformin action is multi-directional, and involves inhibition of gluconeogenesis and glycogenolysis and an increase in tissue sensitivity to insulin through elevating peripheral uptake and tissue glucose consumption [15]. The pharmacological activity of metformin is not limited to metabolic activity [16]. Importantly, metformin was found to significantly decrease diabetes-related death and affect beneficially the cardiovascular system through improvement of endothelium function, reduction of smooth muscle proliferation, and decrease in reactive oxygen species (ROS) production. Some studies report also the effects of metformin on platelet and plasma hemostasis [17,18]. However, the data are limited, sparse and unstructured [16]. Another important issue justifying the need to modify the structure of the biguanide skeleton is the unfavorable pharmacokinetics of the drug. Our previous studies reporting modification of metformin into nitro-benzenesulfonamides or halogenated benzenesulfonamides have found that these compounds are characterized by more profound anti-coagulation properties than the parent drug, metformin [19,20]. Therefore, this study aims at synthesis and biological evaluation of novel sulfonamide-based derivatives of metformin with alkyl substituents in benzene ring in order to obtain more pronounced anti-coagulant properties.

**Scheme 1:**
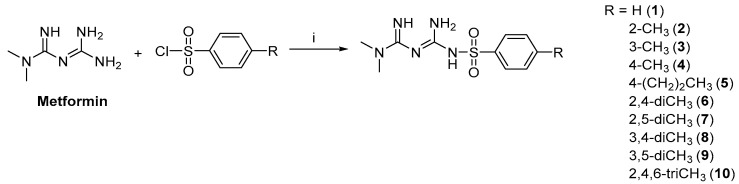
Synthetic route for metformin sulfonamide derivatives; (**i**) CH_2_Cl_2_/DMF, MW, 100 °C, 15 min, 42–77%.

In the present study, 10 novel promising sulfonamide-derived biguanides (Scheme 1) are studied for their glucose-utilization and anti-coagulant properties. Noteworthy, this is the first report to consider the hemocompatibility and ability to affect the function of human endothelial cells compared to parent drug, metformin. Based on the glucose-lowering properties and safety profile as well as anti-coagulant effects, five of the most promising compounds were selected for further in-depth studies, including clot formation and fibrinolysis test and activity of coagulation Xa factor. The effects of these compounds on the release of tissue plasminogen activator (t-PA) from human endothelial cells is also studied. Furthermore, the compounds are examined for intracellular ROS production in endothelial cells. These results show that chemical modification of metformin into sulfonamides can result in obtaining compounds characterized by bi-directional activity, including glucose utilization and anti-coagulant properties.

## 2. Results

### 2.1. Synthesis and Stability of Metformin Derivatives

Sulfonamide derivatives **1**–**10** of metformin were obtained, as was previously described, by coupling commercial sulfonyl chlorides with basic metformin to yield the final compounds with moderate to good yields (Scheme 1) [20,21].

The results of stability studies are shown in Table 1. All compounds were stable in buffer pH 7.4, except for compound **10**, human plasma and rat liver subcellular fractions (S9). According to these data, compound **10** was unstable in aqueous solution but was stable in the presence of human plasma or rat liver subcellular fraction most likely due to the unspecific protein binding in these biological media.

### 2.2. Glucose Utilization Assay

**Figure 1:**
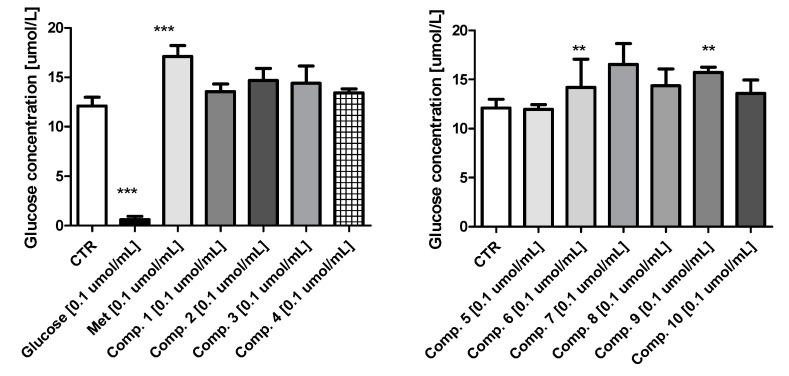
Effects of metformin and synthesized compounds **1**–**10**, and ᴅ-glucose on the 2-NBDG (2-(N-(7-Nitrobenz-2-oxa-1,3-diazol-4-yl)Amino)-2-Deoxyglucose) uptake in human umbilical vein endothelial cells (HUVEC) cells. The cells were treated with the tested compounds for 24 h prior to the experiments. On the day of the experiment the cells were glucose-starved for 2 h, then incubated with insulin (30 min), and in sequence with 2-NBDG for 45 min. The results are presented as mean ± SD, *n* = 4–6. Significant differences in glucose uptake are denoted with asterisk; ** *p* < 0.01, *** *p* < 0.001.

Glucose utilization in human umbilical vein endothelial cells (HUVEC) was determined using a fluorescent glucose analog (2-NBDG; 2-(N-(7-Nitrobenz-2-oxa-1,3-diazol-4-yl)Amino)-2-Deoxyglucose), which is also an indicator of cell viability. The cells were treated with synthesized compounds (**1**–**10**) at 0.1 and 0.3 μmol/mL in DMEM medium for 24 h, and then starved for 2 h, followed by incubation with insulin and 2-NBDG (Figure 1, Figure S1, Supplementary Materials). The uptake of 2-NBDG was inhibited by D-glucose (0.6 ± 0.3 µmol/L versus 12.8 ± 0.4 µmol/L for control). Metformin, a reference compound, was found to significantly increase glucose uptake in HUVECs (17.9 ± 1.0 µmol/L at 0.3 μmol/mL versus 12.8 ± 0.4 µmol/L for control). Comparable effects were reported for compounds **6**, **9** and **10** at 0.1 μmol/mL. All monomethyl derivatives (**2**–**4**) were found to increase glucose uptake; however, the changes were statistically insignificant. For instance, compound **2** at 0.1 μmol/mL led to glucose uptake of 14.7 ± 1.2 μmol/L vs. 11.4 ± 0.7 μmol/L in the case of control (NS, *p* > 0.05). Other sulfonamides did not exert any effects on glucose uptake in HUVECs.

### 2.3. Basic Coagulation Studies

The effects of synthesized sulfonamides **1**–**10** on the basic coagulation parameters are shown in Table 2. Compound **1,** which does not have any substituent in the aromatic ring, neither affects the extrinsic and intrinsic coagulation pathway nor fibrin polymerization over the entire concentration range. Among mono-methyl derivatives (**2**, **3**, **4**), compound **4** with *p*-methyl substituent affects all measured parameters, Activated Partial Thromboplastin Time (APTT) (0.6–1.0 μmol/mL), Prothrombin Time (PT) (1.0 μmol/mL) and Thrombin Time (TT) (0.6 and 1.0 μmol/mL). Among dimethyl derivatives (**6**–**9**), compound **6** with 2,4-dimethyl groups also prolonged extrinsic coagulation pathway (13.1 ± 0.6 s vs. 11.9 ± 0.9 s for control, *p* < 0.05), intrinsic coagulation pathway (53.6 ± 7.0 s vs. 38.0 ± 4.8 s for control, *p* < 0.05) and the process of fibrin polymerization (31.1 ± 3.2 s vs. 14.5 ± 1.5 s for control, *p* < 0.05). Interestingly, derivative **8** with 3,4-dimethyl groups also affected all coagulation parameters; however, PT was not statistically significant (*p* > 0.05). Other dimethyl derivatives contributed to a significant increase in APTT and TT parameters. The introduction of the longer chain (C_3_H_7_^−^) in the aromatic ring did not lead to obtaining more profound anti-coagulation properties in comparison to methyl derivatives. Similarly, three methyl groups in the benzene ring led to significant prolongation of APTT and TT without affecting PT and International Normalized Ratio (INR).

### 2.4. Red Blood Cells Lysis Assay and Morphology

**Figure 2:**
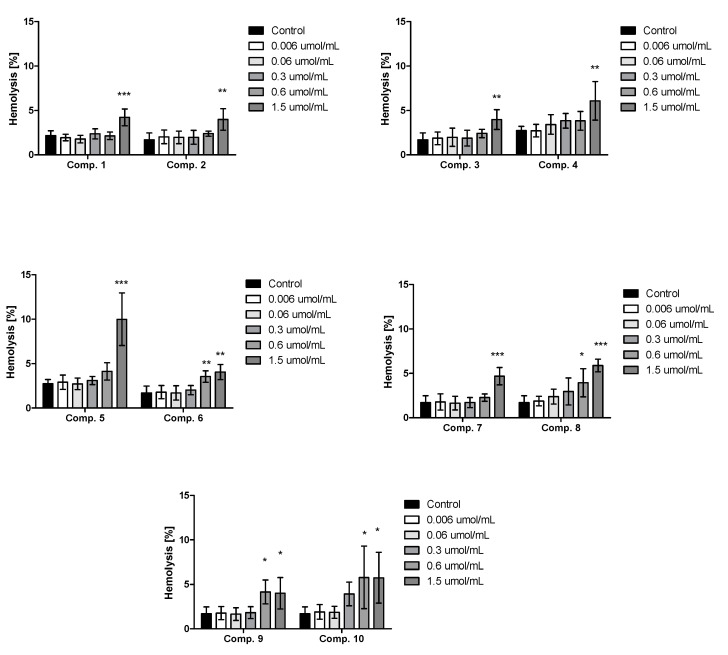
Effects of synthesized compounds **1**–**10** on the hemolysis of erythrocytes. Positive control was Triton X-100, which constituted 100% hemolysis. The results are presented as mean ± S.D.; *n* = 4, * denotes significant differences compared to control (* *p* < 0.05; ** *p* < 0.01; *** *p* < 0.001). Exposure to all compounds at 1.5 μmol/mL contributed to a significant increase in erythrocyte hemolysis; however, in most cases this value did not exceed 5%. In our previous study [22], metformin was not found to significantly affect RBC (red blood cell) hemolysis.

An important element of the examination of new compounds is to determine their effects on the integrity of the erythrocyte membrane. This study was performed spectrophotometrically by measuring the amount of hemoglobin released from RBC (red blood cell) suspension. The effects of synthesized sulfonamide derivatives of metformin (**1**–**10**) on the integrity of the erythrocyte membrane are presented in Figure 2. The conducted analyzes showed that all compounds at the highest tested concentration (1.5 µmol/mL) statistically significantly increased the percentage of hemolyzed erythrocytes. However, in most cases the degree of RBC hemolysis accounted for approximately 5–6%. Only a derivative with a propyl chain in the aromatic ring (compound **5**) at a concentration of 1.5 µmol/mL contributed to hemolysis reaching the value of 9.99 ± 2.95%. In our previous study, metformin did not exert any effect on the integrity of erythrocyte membrane over the entire concentration range (0.006–1.5 µmol/mL) [22]. For instance, one-hour incubation of human RBC with metformin at 1.5 µmol/mL resulted in 1.81 ± 0.78% hemolysis [22].

**Figure 3:**
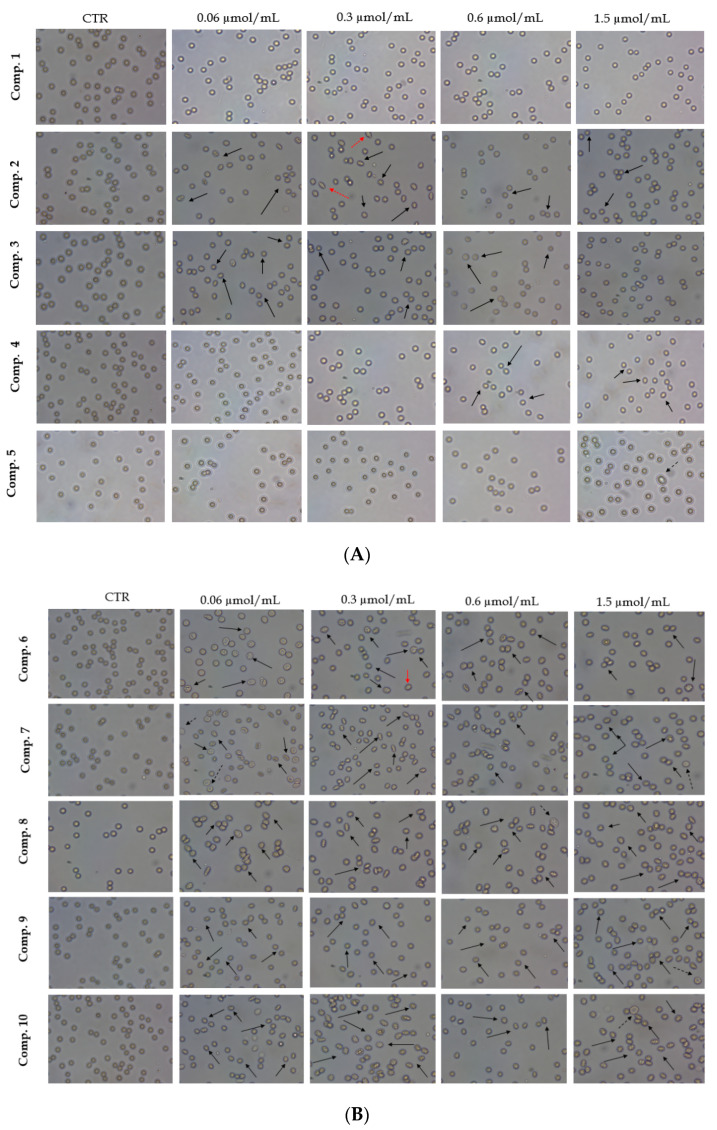
(**A**,**B**). Effects of synthesized compounds **1**–**10** on erythrocytes morphology. Representative phase-contrast images are shown (magnification of 400 times), echinocytes are marked with black arrows, ovalocytes are marked with black dashed arrows and stomatocytes are marked with red arrows. In these studies, metformin was used as a reference compound. It was found to induce the formation of echinocytes. At higher concentrations (1.5 μmol/mL), RBC membrane deformability could also be observed which might be attributed to eryptosis (Figure S2, Supplementary Materials).

Erythrocyte morphology after treating with the tested compounds (**1**–**10**) was assessed with the use of a phase contrast microscope (Figure 3A,B). Compound **1** without any substituents in the aromatic ring did not contribute to changes in the morphological structure of erythrocytes during 1-h incubation over the entire concentration range (0.06–1.5 µmol/mL). Compound **5** demonstrated a similar effect; only single ovalocytes were observed at a concentration of 1.5 µmol/mL. Compounds with two and three methyl groups (**6**–**10**) led to the formation of echinocytes. In addition, incubation with compound **7** at a concentration of 0.06 µmol/mL led to the formation of single ovalocytes. Compounds **2** and **3** showed a tendency to echinocyte formation; however, single stomatocytes and ovalocytes were also recognized. Importantly, the percentage of echinocyte forms after 1-h incubation with compound **3** at a concentration of 1.5 µmol/mL was noticeably reduced than at its lower concentrations. In summary, the microscopic analysis showed a small interaction of the studied metformin analogues with the red blood cell membrane since mainly physiological transformation of discocytes into echinocytes and stomatocytes was observed.

### 2.5. Viability of HUVEC Cells

The effects of the compounds **1**–**10** on the viability of human endothelial cells (HUVEC), were determined using a WST-1 assay. The cells were stimulated with the test compounds at various concentrations, ranging from 0.006 µmol/mL to 1.5 µmol/mL for 24 h. The results are presented in Table 3. Among the tested compounds, derivatives **1**, **4** and **5** appear to have the lowest effect on the viability of HUVECs. Viability of the cells after 24 h incubation with compounds **1**, **4** and **5** at the concentrations of 1.5 µmol/mL was 46.8 ± 1.8%, 40.0 ± 8.6% and 47.7 ± 7.3%, respectively. Dimethyl and trimethyl derivatives (**6**–**10**) exerted greater inhibitory effects on HUVECs growth (Table 3).

The effects of compounds **1**–**10** on HUVEC viability were also monitored using light and phase-contrast microscopy. The effects of two representative concentrations are depicted in Figure S3 (Supplementary Materials). The images were taken after 24 h of co-treatment of the cells with the tested compounds. In the case of constant concentration (0.2 µmol/mL), morphological examination of HUVECs did not reveal any substantial compound-mediated changes.

### 2.6. Endothelial Cell Migration in Real Time

The potential of metformin and its derivatives to reduce endothelial cell migration was investigated using an in vitro wound healing assay. The assay was performed with the JuLiStage system, which allows to visualize cell growth images and curves in real time. The cells were seeded on 96-well plates for 24 h; a wound was made, and then it was co-treated with various concentrations of synthesized compounds, and metformin as a reference. The ability of compounds to affect HUVEC cell migration was monitored microscopically over 24 h of stimulation. The potential for metformin derivatives **1**–**10** to attenuate cell migration is presented in Table S1 (Supplementary Materials). The results are expressed as the width of scratched wound calculated using provided JuLiStage software (NanoEnTek, Waltham, MA, USA).

**Figure 4:**
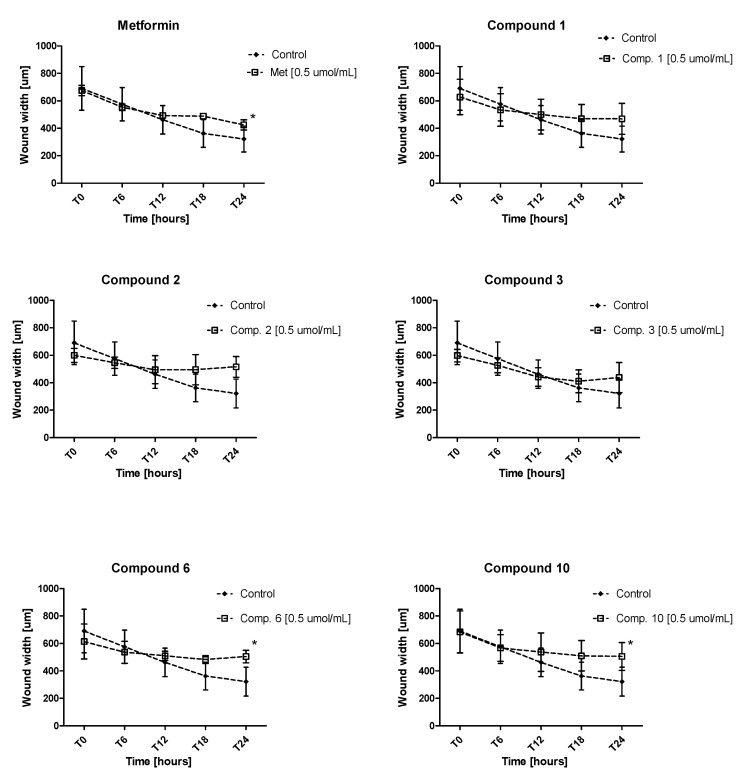
Inhibition of cell migration in the presence of selected compounds (metformin, compounds **1**–**3**, **6**, **10**). HUVEC cell migration was evaluated using wound healing assay performed in the JuLiStage system. Graphs depict changes of the wound width [µm] during 24 h in the absence (control) and in the presence of examined compounds at 0.5 μmol/mL. The results are presented as mean ± SD (*n* = 4–8). * *p* < 0.05 compared with control.

Metformin was found to moderately modulate the cell migration, indicated by an increase in the width of the wound in comparison with control at both tested concentrations (0.1 and 0.5 μmol/mL) (Figure 4, Table S1). However, significant differences were reported only for 0.5 μmol/mL after 24-h incubation. Similar results were obtained for synthesized compounds **1**–**10** (Table S1). Generally, the greatest effect on the wound width was observed after 24 h of incubation (Figure 4). Compounds **6** and **10** at a concentration of 0.5 μmol/mL contributed to significant inhibition of HUVECs migration after 24-h incubation (*p* < 0.05). Other compounds at 0.5 μmol/mL have also led to reduced migration of HUVECs after 18 and 24-h incubation. However, these effects were not statistically significant.

Figure S4 (Supplementary Materials) shows representative images of wound closure at the starting point, 6, 12, 18 and 24 h of stimulation with metformin and compound **10** at the concentration of 0.5 µmol/mL. Metformin did not contribute to significant changes in the morphology of HUVECs, but after 24-h incubation, a significant decrease in cell migration was reported. In the case of compound **10**, severe changes in the morphology of HUVEC cells, manifested by cell shrinkage, were observed upon the addition of compound (T_0_ point). However, further incubation restored the physiological shape of the cells after 6 h.

### 2.7. Clot formation and Lysis Test (CL-Test)

Based on the results of basic coagulation tests, and the effects on integrity of RBCs membrane, compounds **2**, **3**, **4**, **6** and **10** were examined for further in-depth insight into anti-coagulation potential using the CL-test. This assay allows to estimate in vitro effects of the compounds on the overall potential of clot formation and fibrinolysis (CL_AUC_) as well as several kinetic parameters of the clot formation phase, clot stabilization and fibrinolysis [22].

#### 2.7.1. Overall Potential of Clot Formation and Fibrinolysis (CL_AUC_)

**Figure 5:**
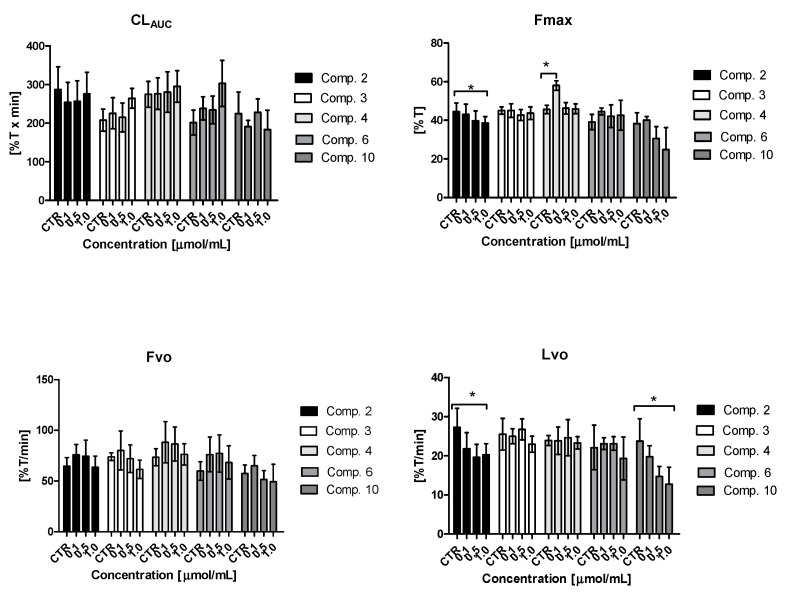
Effects of sulfonamides **2**, **3**, **4**, **6**, and **10** on the overall potential of clot formation and fibrinolysis (CL_AUC_), and selected parameters of clot formation and fibrinolysis process: maximum clotting (Fmax); initial plasma clotting velocity (Fvo); initial clot fibrinolysis velocity (Lvo). The results are presented as mean ± standard deviation (SD), *n* = 5–6. * denotes significant difference (*p* < 0.05) between the sample with tested compound and respective controls. In our previous study metformin was found to not affect the overall potential of clot formation and fibrinolysis (CL_AUC_) over the entire concentration range. Metformin also did not influence the kinetic parameters of the process (Fmax, Fvo, Lvo constans) [22].

All examined compounds did not appear to significantly affect the overall potential of clot formation and fibrinolysis (CL_AUC_ constant expressed as the area under the curve of clot formation and fibrinolysis, Figure 5, Tables S2–S6, Supplementary Materials). However, compounds **2**, **3** and **10** at 1.0 μmol/mL significantly prolonged the total time of clot formation and fibrinolysis (↑ T, Tables S2–S6, Supplementary Materials).

#### 2.7.2. Kinetic Parameters of Clot Formation Phase

With respect to the first phase of the examined process of clot formation, we found that compounds **3**, **4** and **6** induced significant changes in the length of thrombin time (↑ Tt); however, the other compounds also tended to prolong Tt. Incubation of human plasma with compound **2** at 1.0 μmol/mL was associated with a reduction of maximum clotting (↓ Fmax) (Figure 5), which can be attributed to their effects on the structure of the formed clot. Importantly, the compounds did not affect significantly other parameters of clot formation such as plasma clotting time (Tf constant), and the initial velocity of plasma clotting (Fvo constant). Most of the compounds, apart from compound **2** at 0.5 and 1.0 μmol/mL did not alter the area under the curve values for plasma clot formation, which suggests that these compounds do not interfere with the process of clot formation, and are not associated with any increased risk of clot formation.

#### 2.7.3. Kinetic Parameters of Clot Stabilization Phase

Compounds **2** and **3** at 1.0 μmol/mL, and compound **6** at 0.5–1.0 μmol/mL significantly affected the second phase i.e., the clot stabilization phase by prolonging clot stabilization time (↑ Tc) (Tables S2–S6, Supplementary Materials). In the case of compounds **3** and **6**, this alteration contributed to the increased area under the clot formation phase (↑ Sc). In turn, compounds **4** and **10** did not change clot stabilization time (Tc constant) over the entire concentration range.

#### 2.7.4. Kinetic Parameters of Fibrinolysis Phase

The examined compounds also influenced the third phase of the process, i.e., fibrinolysis. Changes in maximum fibrinolysis (Lmax) reflect those observed for Fmax. For instance, compound **2** at the concentration of 1.0 μmol/mL significantly decreased Fmax and Lmax (Table S2, Supplementary Materials). These changes suggest complete lysis of previously-formed clots. Importantly, compounds **3**, **4** and **6** do not affect other parameters of fibrinolysis, e.g., initial velocity of fibrinolysis (Lvo constant), which means that the previously formed clots do not persist longer than in the case of control samples. With regard to fibrinolysis time (Tl), statistically significant changes were observed for the highest concentrations (0.5 and 1.0 μmol/mL) of compounds **2** and **10** (↑ Tl). These compounds also contributed to a significant decrease in the initial velocity of fibrinolysis (↓ Lvo) (Figure 5).

### 2.8. Factor X Activity

**Figure 6:**
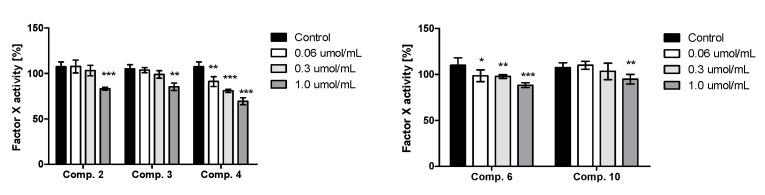
Effects of selected compounds (**2**, **3**, **4**, **6** and **10**) on the activity of factor X (data are presented as mean ± SD; *n* = 4–5) after 1-min incubation with plasma deficient factor X; final volume 210 µL. The asterisk denotes a statistically significant difference between the samples treated with compounds and respective controls; * *p* < 0.05, ** *p* < 0.01, *** *p* < 0.001. All tested compounds at the highest concentration decreased the activity of factor X.

The applied method for the determination of FX activity is based on the observed changes in plasma coagulation time (expressed as PT), which is proportional to the activity of factor X in the tested plasma. Figure 6 depicts the effects of selected sulfonamides on the activity of factor X (FX%). All tested compounds contributed to the significant decrease in FX activity. Simultaneous measurements of PT showed that compounds significantly prolonged this parameter. For instance, compound 2 at 1.0 μmol/mL contributed to increased PT in comparison with control samples (19.8 ± 0.3 s versus 18.3 ± 0.3 s, *p* < 0.001). Importantly, metformin did not contribute to the changes in FX activity (e.g., 89.50 ± 10.75% at 0.6 μmol/mL versus 87.75 ± 13.57% for control (*p* > 0.05) [24].

### 2.9. Tissue Plasminogen Activators Release from Endothelial Cells

The effects of selected sulfonamides on the release of t-PA from HUVEC cells is presented in Table 4. All tested compounds exhibited the same pattern of influence regarding t-PA release; all examined compounds at both concentrations significantly reduced the amount of t-PA in cells supernatants. For instance, derivative **2** at 0.1 and 0.3 µmol/mL significantly decreased t-PA concentration (1553.5 ± 305.2 pg/mL at 0.1 µmol/mL and 544.5 ± 80.9 pg/mL at 0.3 µmol/mL vs. 2883.6 ± 138.7 pg/mL for control, *p* < 0.001) (Table 4).

Metformin was found to significantly increase t-PA release from HUVECs, for instance, incubation of metformin at 0.3 μmol/mL with the cells resulted in 2652.3 ± 105.3 pg/mL t-PA released in comparison with control (2297.8 ± 89.4 pg/mL) [19].

### 2.10. Intracellular ROS Generation

**Figure 7:**
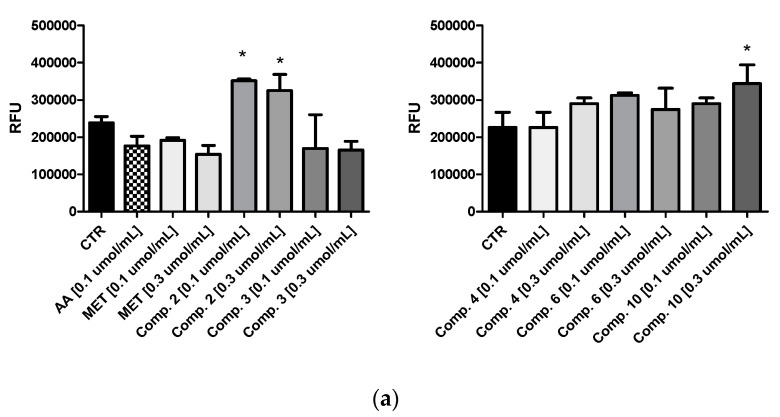

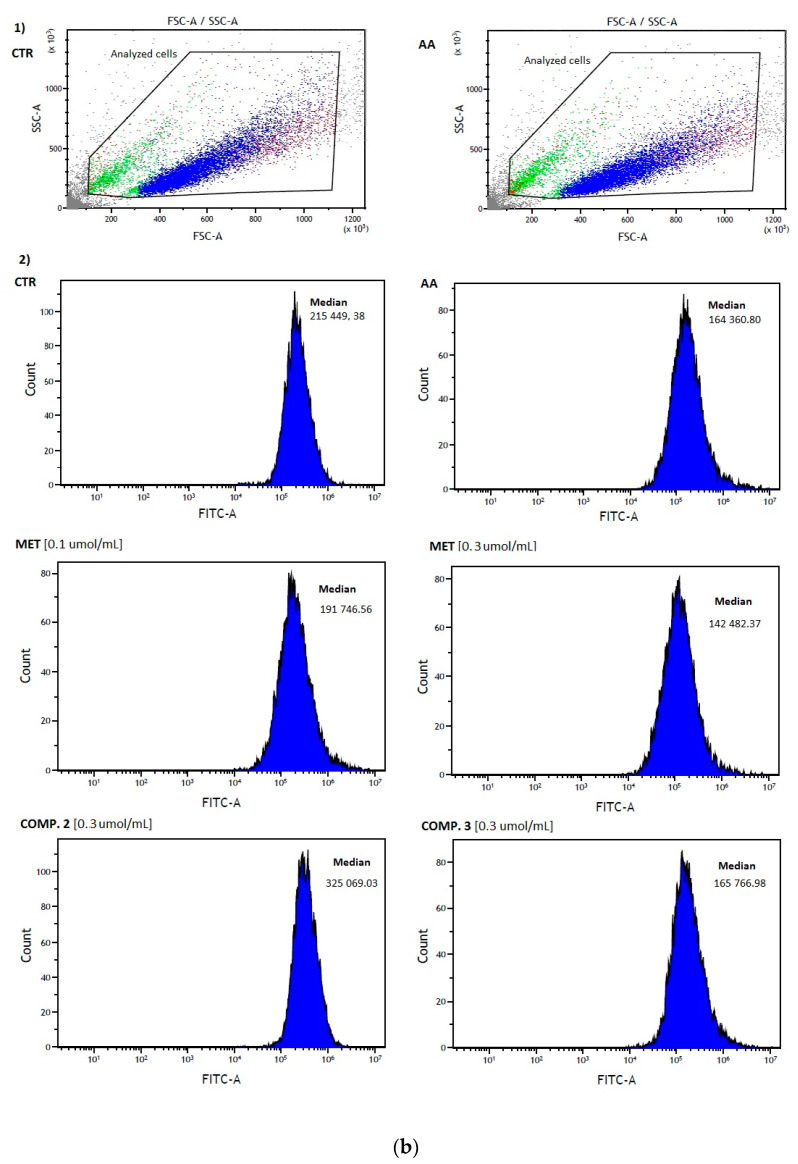
Effects of selected biguanides on the production of intracellular reactive oxygen species. The experiments were performed using 2′,7′-dichlorodihydrofluorescein diacetate (H_2_DCFDA), a fluorescent indicator for ROS in cells. The fluorescence of stained HUVECs was measured by flow cytometry. (**a**) Summary of the effects of metformin, compound **2**, **3**, **4**, **6** and **10** on the H_2_DCFDA fluorescence in viable HUVEC cells. The results are presented as median ± range, *n* = 3). The asterisk denotes a statistically significant difference in comparison to control, * *p* < 0.05. Ascorbic acid (**AA**) was used as a reference compound; (**b**) Flow cytometry analysis of the intracellular fluorescence in HUVECs. (**a**) Representative histograms of unstimulated HUVECs (control), and HUVECs treated with ascorbic acid (0.1 µmol/mL)—forward and side scatter plot of HUVECs; the analyzed cells are gathered in black frame. (**b**) Representative cytograms of analyzed cells treated with metformin (0.1 and 0.5 µmol/mL), and compounds **2** and **3** (0.5 µmol/mL). Median fluorescence intensity of analyzed cells is indicated in every cytogram.

In this paper we also evaluated the influence of synthesized selected sulfonamides on the intracellular generation of ROS in HUVEC cells. Data regarding the effects of tested biguanides together with metformin as a reference drug on the ROS generation in HUVECs are given in Figure 7. Both tested concentrations of metformin decreased ROS production in HUVECs after 24 h incubation in comparison to control cells. Metformin at a concentration of 0.3 µmol/mL caused a 21.2% reduction in the amount of ROS produced compared to the control samples. However, these changes were not statistically significant in comparison to unstimulated cells (Figure 7a,b). The results obtained for ascorbic acid, the reference compound, were comparable to those received for metformin. Compounds **3**, **4** and **6** at both tested concentrations did not appear to influence ROS production in HUVECs. Adversely, compound **2** at 0.1 and 0.3 µmol/mL and compound **10** at 0.5 µmol/mL significantly increased ROS generation, measured as increased fluorescence in comparison to control samples.

## 3. Discussion

To combat the pathogenesis of multifactorial illnesses, interventions and therapy need to be multidirectional and highly specific. Therefore, it has been claimed that the modulation of several drug targets with the aid of a poly-pharmacological approach is necessary to achieve desired therapeutic effects in the treatment of multi-factorial diseases [25]. The multi-target strategy has become one of today’s most promising drug discovery areas, particularly in developing medicines against complex diseases [25]. The review of FDA-approved new drugs from 2000 to 2015 [26] supports the idea that poly-pharmacology is particularly useful for treating diseases with complex etiology, including inflammation, cancer and metabolic syndrome [27]. It has been accepted that simultaneous influence of multiple targets is required to manage diseases with complex pathology. This paradigm has led to the development of new strategies aiming at discovery and development of drugs against metabolic disorders. Using PPARγ (peroxisome proliferator-activated receptor) agonists for the treatment of metabolic syndrome, which is a multifaceted health problem, is a striking example of the applicability of multi-target drugs [28].

Diabetes is one example of a multi-factorial disease, and metabolic abnormalities associated with it are not only linked with elevated glucose levels but also hemostatic disturbances, which include all three phases of coagulation, including vascular, platelet and plasma hemostasis. It has been found that individuals suffering from diabetes are prone to develop hypercoagulability and hypofibrinolysis. Importantly, circa 80% of T2DM subjects die due to thrombotic events. Of this number, 75–80% of deaths occur due to cardiovascular events [29]. Vascular endothelium is primary tissue exposed to negative effects of hyperglycemia. An increased glucose level contributes to endothelial injury and dysfunction, leading to the increased permeability and vasodilation of blood vessels. Metabolic disorders and hyperglycemia occurring in diabetes significantly affect the morphology, function and activation of platelets [30]. With respect to plasma coagulation, the most frequently occurring changes include increased activity of certain coagulation factors, such as FVII, FVIII and fibrinogen, and hypercoagulability markers, including thrombin-antithrombin complex (TAT) and fibrinopeptide A (FPA) [29].

Considering the above-mentioned disorders of hemostasis associated with diabetes, it is justified to search for such agents that have both hypoglycemic and anti-coagulant effects. Metformin is just one of those promising drugs that has multidirectional effects in T2DM. Apart from glucose-lowering properties, metformin exerts numerous additional activities that have been a topic of several reviews [31,32,33]. Properties regarding cardiovascular effects, coagulation and endothelial function are particularly important [16]. Recently, Sardu et al. [34] reported that therapy with metformin may decrease the risk of cardiovascular event in pre-diabetes patients due to the reduction of coronary endothelial dysfunction. In addition, scientists are also interested in the anti-cancer effect of metformin [35,36]. For instance, Della Corte et al. [37], found metformin potentiates the anti-tumor activity of MEK inhibitors (selumetinic and pimasertib) in human LKB1-wilde-type non-small cell lung cancer (NSCLC cell line) through glioma-associated oncogene homolog 1 (GLI1) downregulation and by reducing transcription of metalloproteinase-2 and 9 (MMP-2 and MMP-9).

Despite all these beneficial pharmacodynamic properties, metformin is characterized by unfavorable pharmacokinetic properties, including moderate and slow intestinal absorption, which results in 50–60% bioavailability. These properties stem from the chemical structure of metformin, *N*,*N*-dimethylbiguanide hydrochloride, which is highly hydrophilic (logP octanol: water = −2.6), and exists in a protonated form at physiological pH [38]. Importantly, the drug is unable to diffuse passively through the cellular membrane, and its cellular uptake is based on transporters, mainly organic cation transporters [31]. Therefore, the impaired expression, function or polymorphism of these carriers affect metformin oral absorption, distribution and elimination, as well as its biochemical effects in humans.

Taking the associations between diabetes and hypercoagulability as well as pleiotropic activity of metformin into consideration, there is a need to design and synthesize novel analogs of biguanide with improved bioavailability. One of these strategies appears to be chemical transformation of biguanide backbone into sulfonamides, which allows to obtain molecules with promising anti-coagulant activity [19,20]. The choice of sulfonamides seems to be well justified especially in the light of the results of the review by Apaydin and Torok, who claimed that the use of sulfa-drugs has been extended to targeting complex diseases including other CNS disorders, diabetes and various cancers and tumors [3]. The structural versality of sulfa-drugs and well-established available information make them promising candidates for development of more effective alternatives to currently approved drugs.

Herein, we present the synthesis and biological evaluation of 10 novel sulfonamide-based analogs of metformin. In the metformin scaffold, one terminal nitrogen atoms was modified with benzenesulfonamide groups substituted with alkyl groups. This strategy was aimed to obtain novel unreported agents with glucose-lowering and anti-coagulation properties.

Metformin glucose-lowering properties can be attributed to several mechanisms. One of them is related to its action on glucose metabolism, specifically as an inhibitor of hepatic gluconeogenesis by blocking mitochondrial glycerophosphate dehydrogenase, and as a consequence, reducing glucose formation from lactate and glicerol [39]. In addition, metformin was proven to increase peripheral glucose uptake, and reduce glucose absorption from the gastrointestinal tract [40]. Within this study, we have found that most of dimethyl derivatives significantly improved glucose utilization in endothelial cells, while monomethyl derivatives (**2**–**4**) were found to slightly elevate glucose uptake; however, these changes were not of statistical significance. The authors are aware that these experiments are only preliminary, and evaluate only one possible mechanism of glucose-lowering activity. In addition, it would also be vital to assess the effect of these compounds using different cell models more related to the glucose metabolism, such as hepatocytes or pancreatic cells, and subsequently in in vivo pre-clinical studies.

The basic coagulation studies showed that derivative **4** with *p*-methyl substituent, and **6** with two methyl groups at **2** and **4** position in the aromatic ring affected both the intrinsic and extrinsic coagulation pathway. In addition, both compounds significantly prolong the process of fibrin polymerization, expressed as increased TT. All other synthesized compounds contributed to the significant increase in APTT and TT. Results presented in this study as well as those obtained in a previous one [22], in which metformin did not appear to affect APTT and PT, revealed that benzenesulfonamides with methyl substituents in the aromatic ring significantly affected the intrinsic coagulation pathway and the process of fibrin polymerization, and thus, may exert beneficial effects on plasma hemostasis. Importantly, also other sulfonamide-based metformin analogs, such as those with an *o*- or *p*-nitro substituent in the aromatic ring exert highly beneficial anti-coagulation properties, manifested by prolonged PT and APTT [41]. These results are important, since recent studies have shown that shortened APTTs may reflect pro-coagulant imbalances with increased levels of coagulation factors. As stated by Lippi et al. [42], APTT might identify diabetic patients at major risk of thrombosis. Therefore, the properties of synthesized compounds towards increasing APTT might be of vital importance upon the commencement of pre-clinical studies, and suggest more profound anti-coagulant properties of synthesized compounds than that of the parent drug, metformin.

On the base of the obtained results of basic coagulation studies, we attempted to conduct additional experiments that allow us to comprehensively characterize anti-coagulant properties of selected compounds. Several compounds were chosen for further studies, which involved making measurements of factor X activity. The experiments showed that all selected compounds (**2**, **3**, **4**, **6** and **10**) significantly decreased the activity of FX. Importantly, compounds **4** and **6** contributed to a significant decrease in FX activity over the entire concentration range which can explain the effects of these compounds on measured PT and APTT. In conclusion, the results of FX experiments confirm the anti-coagulant properties of synthesized metformin analogs. Thus, these data give evidence on more pronounced anti-coagulant activity of examined compounds in comparison with those of metformin. 

Compounds **2**, **3**, **4**, **6** and **10** were further studied using a multiparametric CL-test, which allows to measure the overall potential of clot formation and fibrinolysis and to calculate a number of kinetic parameters of this process. Importantly, the incubation of human plasma with examined compounds did not result in significant changes in the overall potential of clot formation and fibrinolysis (CL_AUC_ constant). This is an important finding in view of the results of He et al. [43], who found that the greater overall hemostasis potential (OHP), which constitutes an equivalent to CL_AUC_, correlates with an increased risk of cardiovascular events. Regarding the kinetic parameters of the clot formation phase, we have found that the tested compounds have a tendency to prolong thrombin time (↑ Tt), confirming the results obtained in the TT test. Both these experiments imply that the tested compounds affect the process of fibrin polymerization. Since the compound does not change the initial clot formation velocity (Fvo constant), we presume that the examined compounds do not affect the activity of thrombin. Importantly, most of the compounds except for compound **2**, do not alter the structure of clot (Fmax constant), and do not affect the area under the curve of clot formation. Therefore, we can state that the sulfonamide-based analogs of metformin are not associated with an increased risk of clot formation. Of all tested compounds, derivative **4** did not appear to affect the parameters of fibrinolysis, and therefore, is not related with the risk of hypo-fibrinolysis, a process frequently found in diabetic patients [9]. Collectively, we presume that the application of examined compounds might be regarded as having no risk associated with plasma hemostasis since they mostly do not affect the process of clot formation. With regards to fibrinolysis, the neutral potential was shown by compound **4** with *p*-methyl substituent in the aromatic ring.

To further characterize the biological properties of a series of metformin analogs we have performed experiments using HUVEC cells. These endothelial cells were chosen due to their numerous functions, including maintenance of proper vessel homeostasis and blood flow, regulation of thrombosis and clotting, vessel growth and angiogenesis. In addition, the assessment of the effects of novel compounds on HUVECs is vital, since they constitute an inner layer of the vascular wall, and they have a direct contact with a substance that is in the bloodstream. It also participates in maintaining the balance between pro-coagulant and anti-coagulant factors [44]. The analysis of structure-activity relationship allowed to assume that the para position of substituent in the aromatic ring is associated with greater safety expressed as higher viability of cells. Monomethyl derivatives are characterized by relatively lower effects on HUVECs growth while dimethyl derivatives (**6**–**9**) and trimethyl derivative (**10**) interact stronger with endothelial cells.

The effects of synthesized compounds on HUVECs were also evaluated using the wound healing assay, conducted with the application of the JuLiStage system which enables real-time monitoring of live cells. Our studies have shown that metformin, after 24-h stimulation, significantly decreased HUVECs migration. Other scientists revealed that metformin was also able to inhibit HUVECs migration [45]. Importantly, metformin also suppresses proliferation and migration in cardiac fibroblasts. In the current study, all examined sulfonamides exerted an effect that was similar to that of metformin. However, statistically significant results were obtained only for compounds **6** and **10**. The outcomes of migration assay give crucial information on the valuable properties of examined metformin analogs, since pathological migration is a major factor in atherogenesis [46].

Reactive oxygen species (ROS) are critically important chemical intermediates in biological experiments because they participate in numerous physiologically relevant functions and demonstrate their frequent pathologically detrimental effects. Therefore, it is important to quantify their level in biological samples [47]. The anti-oxidative properties of metformin were examined in multiple studies. For instance, An et al. [48], evaluated the influence of metformin on fluctuating glucose-induced endothelial dysfunction. Obtained results indicate that metformin has protective properties towards endothelial cells against oxidative stress. The results of flow cytometry studies of ROS showed that metformin significantly decreases intracellular ROS production in HUVECs. Compound **3** was also found to reduce ROS generation; however, these changes were statistically insignificant. The fact that metformin is not effective against all oxidative stress inducers should be considered when evaluating antioxidant properties of this drug. For instance, Algire et al. [49], reported that metformin attenuates paraquat-induced elevations in ROS level, and related DNA damage and mutations, but has no effect on similar changes induced by H_2_O_2_. The authors are aware that further in-depth studies are needed to assess the anti-inflammatory properties of sulfonamide-based analogs of metformin, and their effects on NF-κB activity, especially in view of the fact that metformin was found to inhibit of the expression of NF-κB gene, reduce inflammation and eliminate the susceptibility to common diseases such as cancer or atherosclerosis [50].

To sum up, this study describes the synthesis of novel metformin analogs and their biological assessment using experimental in vitro models. Most of the results obtained in these studies seem to be promising, and we proved that sulfonamide-based metformin analogs can be regarded as dual-action drug candidates. The next stage of the research will be to check the effectiveness of selected sulfonamide derivatives using in vivo experimental models.

## 4. Materials and Methods

### 4.1. General Synthetic Materials and Methods

All reactions were performed with reagents obtained from MilliporeSigma (St. Louis, MO, USA) or ThermoFisher Scientific (Waltham, MA, USA). Reactions were monitored by thin-layer chromatography using aluminum sheets coated with silica gel 60 F245 (0.24 mm) with suitable visualization. Purifications by flash chromatography were performed on silica gel 60 (0.063–0.200 mm mesh). ^1^ H and ^13^ C nuclear magnetic resonance (NMR) spectra were recorded on a Bruker Avance 500 spectrometer (Bruker Biospin, Fällanden, Switzerland) operating at 500.13 MHz and 125.75, respectively, using tetramethylsilane as an internal standard. Not all pH-dependent protons of the compounds were observed. ESI-MS spectra were recorded by an Agilent 1260 Infinity LC system coupled with an Agilent 6410 triple quadrupole mass spectrometer with an electrospray ionization source (Agilent Technologies, Palo Alto, CA, USA). Over 97% purities were obtained for the final products by an elemental analysis (C, H, N) with a Perkin Elmer 2400 Series II CHNS/O organic elemental analyzer (Perkin Elmer Inc., Waltham, MA, USA).

### 4.2. General Procedure for Synthesis of Sulfenamide Derivatives

Metformin (*N*,*N*-dimethyl imidodicarbonimidic diamide hydrochloride) (1.0 eq.) in 1 M NaOH (1.5 eq.) was stirred at room temperature for 30 min. Water was evaporated in vacuo and the residue was dissolved in MeOH. The solvent was evaporated and the residue was redissolved in cold anhydrous MeOH. NaCl was filtered out of the solution and the filtrate was evaporated to yield basic metformin as a white solid (99%).

Basic metformin (2.0 eq.), and commercial sulfonyl chlorides (1.0 eq.) were dissolved in anhydrous CH_2_Cl_2_/DMF (10:1) in a sealed pressure-rated glass tube and irradiated at 100 °C with a microwave synthesizer (Biotage Initiator+, Uppsala, Sweden) for 15 min. The solvent was evaporated under reduced pressure and the residue was purified by flash column chromatography eluting with 0–10% MeOH in CH_2_Cl_2_ to obtain the compounds **1**–**10** (42–77%). Some of the final products were triturated with diethyl ether, methanol or acetone to improve their purity.

*N*^1^*,N*^1^*-Dimethyl-N*^4^*-benzenesulfonamide-biguanidine* (**1**). Compound **1** was prepared from benzenesulfonyl chloride to yield off-white solid, 0.39 g, (75%). ^1^H NMR ((CD_3_)_2_SO): δ ppm 8.04 (bs, 2H), 7.70–7.75 (m, 2H), 7.56–7.49 (m, 2H), 6.96 (bs, 1H), 6.71 (bs, 1H), 2.91 (s, 6H); ^13^C NMR ((CD_3_)_2_SO): δ ppm 159.83, 158.39, 144.23, 131.33, 128.79 (2C), 125.67 (2C), 36.53 (2C). MS (ESI+) for C_10_H_16_N_5_O_2_S (M + H) +: Calcd 270.33, Found 270.18. Anal. Calcd for (C_10_H_15_N_5_O_2_S *0.1 (CH_3_)_2_CO): C, 44.97; H, 5.64; N, 25.45; Found: C, 45.07; H, 5.20; N, 25.78.

*N*^1^*,N*^1^*-Dimethyl-N*^4^*-(2-methylbenzenesulfonamide)-biguanidine* (**2**). Compound **2** was prepared from 2-methylbenzenesulfonyl chloride to yield off-white solid, 0.34 g, (62%). ^1^H NMR ((CD_3_)_2_SO): δ ppm 8.12 (bs, 2H), 7.85 (d, J = 7.8 Hz, 1H), 7.43 (t, J = 7.5 Hz, 1H), 7.33–7.29 (m, 2H), 6.90 (bs, 1H), 6.60 (bs, 1H), 2.91 (s, 6H), 2.57 (s, 3H); ^13^C NMR ((CD3)2SO): δ ppm 159.64, 158.35, 141.92, 135.94, 132.04, 131.37, 127.11, 125.61, 36.47 (2C), 19.74. MS (ESI+) for C_11_H_18_N_5_O_2_S (M + H) +: Calcd 284.36, Found 284.18. Anal. Calcd for (C_11_H_17_N_5_O_2_S *0.05 (CH_3_)_2_NCHO): C, 46.66; H, 6.09; N, 24.40; Found: C, 46.99; H, 5.97; N, 23.97.

*N*^1^*,N*^1^*-Dimethyl-N*^4^*-(3-methylbenzenesulfonamide)-biguanidine* (**3**). Compound **3** was prepared from 3-methylbenzenesulfonyl chloride to yield off-white solid, 0.25 g, (45%). ^1^H NMR ((CD_3_)_2_SO): δ ppm 8.03 (bs, 2H), 7.59 (s, 1H), 7.55 (d, J = 7.7 Hz, 1H), 7.39 (t, J = 7.5 Hz, 1H), 7.35 (d, J = 7.8 Hz, 1H), 6.93 (bs, 1H), 6.70 (bs, 1H), 2.91 (s, 6H), 2.36 (s, 3H); ^13^C NMR ((CD3)2SO): δ ppm 159.76, 158.34, 144.17, 138.28, 131.89, 128.87, 125.91, 122.82, 36.49 (2C), 20.88. MS (ESI+) for C_11_H_18_N_5_O_2_S (M + H) +: Calcd 284.36, Found 284.10. Anal. Calcd for (C_11_H_17_N_5_O_2_S *0.4 (CH_3_)_2_CO): C, 47.80; H, 6.12; N, 22.84; Found: C, 47.60; H, 5.91; N, 22.69.

*N*^1^*,N*^1^*-Dimethyl-N*^4^*-(4-methylbenzenesulfonamide)-biguanidine* (**4**). Compound **4** was prepared from 4-methylbenzenesulfonyl chloride to yield off-white solid, 0.41 g, (75%). ^1^H NMR ((CD_3_)_2_SO): δ ppm 8.03 (bs, 2H), 7.64 (d, J = 8.1 Hz, 2H), 7.30 (d, J = 7.9 Hz, 2H), 6.93 (bs, 1H), 6.67 (bs, 1H), 2.91 (s, 6H), 2.34 (s, 3H); 13C NMR ((CD_3_)_2_SO): δ ppm 159.82, 158.38, 141.47, 141.32, 129.18 (2C), 125.71 (2C), 36.51 (2C), 20.90. MS (ESI+) for C_11_H_18_N_5_O_2_S (M + H) +: Calcd 284.36, Found 284.18. Anal. Calcd for (C_11_H_17_N_5_O_2_S *0.1 (CH_3_)_2_CO): C, 46.94; H, 6.07; N, 24.22; Found: C, 47.28; H, 5.58; N, 24.30.

*N*^1^*,N*^1^*-Dimethyl-N*^4^*-(4-propylbenzenesulfonamide)-biguanidine* (**5**). Compound **5** was prepared from 4-propylbenzenesulfonyl chloride to yield off-white solid, 0.46 g, (76%). ^1^H NMR ((CD_3_)_2_SO): δ ppm 8.05 (bs, 2H), 7.66 (d, J = 8.3 Hz, 2H), 7.31 (d, J = 8.3 Hz, 2H), 6.93 (bs, 1H), 6.69 (bs, 1H), 2.91 (s, 6H), 2.59 (t, J = 7.6 Hz, 2H), 1.58 (sex, J = 7.4 Hz, 2H), 0.89 (t, J = 7.3 Hz, 3H); ^13^C NMR ((CD_3_)_2_SO): δ ppm 159.82, 158.37, 145.84, 141.71, 128.61 (2C), 125.92 (2C), 36.95 (2C), 36.51, 23.87, 13.65. MS (ESI+) for C_13_H_22_N_5_O_2_S (M + H)+: Calcd 312.41, Found 312.28. Anal. Calcd for (C_13_H_21_N_5_O_2_S *0.1 (CH_3_)_2_CO): C, 50.36; H, 6.80; N, 22.08; Found: C, 50.64; H, 6.41; N, 22.22.

*N*^1^*,N*^1^*-Dimethyl-N*^4^*-(2,4-dimethylbenzenesulfonamide)-biguanidine* (**6**). Compound **6** was prepared from 2,4-dimethylbenzenesulfonyl chloride to yield off-white solid, 0.24 g, (42%). ^1^H NMR ((CD_3_)_2_SO): δ ppm 8.11 (bs, 2H), 7.73 (, J = 8.0 Hz, 1H), 7.13 (s, 1H), 7.10 (d, J = 8.0 Hz, 1H), 6.87 (bs, 1H), 6.56 (bs, 1H), 2.91 (s, 6H), 2.52 (s, 3H), 2.30 (s, 3H); 13C NMR ((CD_3_)_2_SO): δ ppm 159.62, 158.34, 141.26, 139.23, 135.76, 132.61, 127.31, 125.95, 36.45 (2C), 20.64, 19.63. MS (ESI+) for C_12_H_20_N_5_O_2_S (M+H)+: Calcd 298.39, Found 298.10. Anal. Calcd for (C_12_H_19_N_5_O_2_O_2_S *0.2 (CH_3_)_2_CO): C, 48.98; H, 6.46; N, 22.66 Found: C, 49.00; H, 6.38; N, 22.35.

*N*^1^*,N*^1^*-Dimethyl-N*^4^*-(2,5-dimethylbenzenesulfonamide)-biguanidine* (**7**). Compound **7** was prepared from 2,5-dimethylbenzenesulfonyl chloride to yield off-white solid, 0.45 g, (77%). ^1^H NMR ((CD_3_)_2_SO): δ ppm 8.11 (bs, 2H), 7.69 (s, 2H), 7.23 (d, J = 7.6 Hz, 1H), 7.20 (d, J = 7.5 Hz, 1H), 6.89 (bs, 1H), 6.61 (bs, 1H), 2.91 (s, 6H), 2.51 (s, 3H), 2.31 (s, 3H); ^13^C NMR ((CD_3_)_2_SO): δ ppm 159.50, 158.29, 141.70, 134.85, 132.74, 131.96, 131.85, 127.55, 36.48 (2C), 20.46, 19.29. MS (ESI+) for C_12_H_20_N_5_O_2_S (M + H) +: Calcd 298.39, Found 298.10. Anal. Calcd for (C_12_H_19_N_5_O_2_O_2_S *0.6 (CH_3_)_2_CO): C, 49.89; H, 6.49; N, 21.08 Found: C, 49.53; H, 6.17; N, 20.96.

*N*^1^*,N*^1^*-Dimethyl-N*^4^*-(3,4-dimethylbenzenesulfonamide)-biguanidine* (**8**). Compound 8 was prepared from 3,4-dimethylbenzenesulfonyl chloride to yield off-white solid, 0.25 g, (43%). ^1^H NMR ((CD_3_)_2_SO): δ ppm 8.03 (bs, 2H), 7.54 (s, 1H), 7.48 (d, J = 7.7 Hz, 1H), 7.25 (d, J = 7.7 Hz, 1H), 6.90 (bs, 1H), 6.67 (bs, 1H), 2.91 (s, 6H), 2.26 (s, 3H), 2.25 (s, 3H); ^13^C NMR ((CD_3_)_2_SO): δ ppm 159.76, 158.35, 141.66, 140.10, 136.84, 129.53, 126.44, 123.18, 36.47 (2C), 19.39, 19.29. MS (ESI+) for C_12_H_20_N_5_O_2_S (M + H) +: Calcd 298.39, Found 298.10. Anal. Calcd for (C_12_H_19_N_5_O_2_O_2_S *0.2 (CH_3_)_2_CO): C, 48.98; H, 6.46; N, 22.66 Found: C, 48.93; H, 6.11; N, 22.43.

*N*^1^*,N*^1^*-Dimethyl-N*^4^*-(3,5-dimethylbenzenesulfonamide)-biguanidine* (**9**). Compound **9** was prepared from 3,5-dimethylbenzenesulfonyl chloride to yield off-white solid, 0.37 g, (63%). ^1^H NMR ((CD_3_)_2_SO): δ ppm 8.03 (bs, 2H), 7.38 (s, 2H), 7.17 (s, 2H), 6.91 (bs, 1H), 6.70 (bs, 1H), 2.91 (s, 6H), 2.32 (s, 6H); ^13^C NMR ((CD_3_)_2_SO): δ ppm 159.73, 158.33, 144.14, 138.07 (2C), 132.60, 123.19 (2C), 36.48 (2C), 20.79 (2C). MS (ESI+) for C_12_H_20_N_5_O_2_S (M + H) +: Calcd 298.39, Found 298.10. Anal. Calcd for (C_12_H_19_N_5_O_2_O_2_S *0.4 (CH_3_)_2_CO): C, 49.45; H, 6.48; N, 21.84 Found: C, 49.24; H, 6.62; N, 21.67.

*N*^1^*,N*^1^*-Dimethyl-N*^4^*-(2,4,6-trimethylbenzenesulfonamide)-biguanidine* (**10**). Compound **10** was prepared from 2,4,6-triisopropylbenzenesulfonyl chloride to yield off-white solid, 0.35 g, (58%). ^1^H NMR ((CD_3_)_2_SO): δ ppm 8.02 (bs, 2H), 6.95 (s, 2H), 6.79 (bs, 1H), 6.53 (bs, 1H), 2.90 (s, 6H), 2.56 (s, 6H), 2.23 (s, 3H); ^13^C NMR ((CD_3_)_2_SO): δ ppm 159.49, 158.22, 139.93, 138.72, 136.93 (2C), 131.18 (2C), 36.43 (2C), 22.40 (2C), 20.30. MS (ESI+) for C_13_H_22_N_5_O_2_S (M + H) +: Calcd 312.41, Found 312.10. Anal. Calcd for (C_13_H_21_N_5_O_2_S *0.1 (CH_3_)_2_NCHO): C, 50.12; H, 6.86; N, 21.97; Found: C, 50.38; H, 6.65; N, 21.59.

### 4.3. Biological Material for Stability Studies

Rat liver S9 fraction was prepared by collecting fresh tissues from the animals in compliance with the European Commission Directives 2010/63/EU and 86/609 and approved by the Institutional Animal Care and Use Committee of the University of Eastern Finland (No. ESAVI/3347/04.10.07/2015). The liver homogenates were prepared by homogenizing freshly-collected rat liver with 50 mM Tris-buffered saline (TBS) (pH 7.4) (1:4 *w*/*v*) using an Ultra Turrax dispersing instrument (X1020, Ystral Gmbh, Dottingen, Germany). The homogenates were centrifuged at 9000 rpm for 20 min at 4 °C and the supernatant was collected. Protein concentrations of both fractions were determined by Bio-Rad Protein Assay using the Bradford method (EnVision, Perkin Elmer, Waltham, MA, USA). Biological material was stored at −80 °C before using.

### 4.4. High-Performance Liquid Chromatography (HPLC) Analyses

Concentrations of metformin derivatives were determined by HPLC. The apparatus consisted of an Agilent 1100 binary pump (Agilent Technologies Inc., Wilmington, DE, USA), a 1100 micro vacuum degasser, an HP 1050 Autosampler and an HP 1050 variable wavelength detector, operated at 235 nm. The chromatographic separations were achieved on an Agilent Zorbax SB-C18 analytical column (4.6 mm × 150 mm, 5 μm) (Agilent Technologies Inc., Wilmington, DE, USA) by using isocratic elution of water containing 0.1% formic acid (pH ca. 3.0) and acetonitrile containing 0.1% formic acid with a ratio of 75:25 (*v*/*v*). The retention times of the compounds were circa 3.7–4.3 min at a flow rate of 1.0 mL/min at room temperature. The lower limit of quantification for the compounds was 1.0 μM. These HPLC methods were also accurate (100 ± 10% of nominal concentration), precise (RSD% < 10%) and selective (no interfering peaks) over the range 1–100 μM.

### 4.5. Stabilities of Metformin Sulfonamide Derivatives

Enzymatic and chemical stabilities of metformin sulfonamide derivatives in rat liver S9 fractions, in human plasma or in Tris buffer (pH 7.4) were determined at 37 °C. The incubation mixtures were prepared by mixing liver S9 fraction (final protein concentration 1.0 mg/mL) with Tris buffer (pH 7.4) or plasma with 10 mmol/L stock solution of studied compounds in DMSO (the final concentration of compounds were 100 µmol/L and the DMSO concentration was 2%). The mixtures were incubated for six hours and the samples (100 µL) were withdrawn at appropriate intervals. The proteins in the samples were precipitated with ice-cold acetonitrile (100 µL) and the samples were centrifuged for 5 min at 12,000 rpm at room temperature. The supernatants were collected and analyzed by the HPLC method described above. In the chemical stability study in Tris buffer, the S9 fractions or plasma were replaced with the same volume of buffer.

### 4.6. Preparation of Biological Material for Basic Coagulology Studies and RBCs Lysis Assay

The experiments on human blood were performed in accordance with Polish national guidelines, and the study protocols were approved by the Bioethics Committee of the Medical University of Lodz (Medical University of Lodz, Poland, approval no. RNN/27/18/KE).

Blood samples were obtained from the Wojewódzki Specjalistyczny Szpital im. Dr W. Biegańskiego w Łodzi (Voivodship Specialized Hospital in Łódź), Poland; the tested material was a remnant of routine diagnostic tests intended for disposal as medical waste. The blood was collected into vacuum tubes filled with 3.2% buffered sodium citrate, and centrifuged (3000 rpm, 10 min, room temperature) with a Micro 22R centrifuge (Hettich Zentrifugen, Tuttlingen, Germany). Red blood cells were separated, washed three times with 0.9% saline and used for the experiments within 24 h. Poor platelet plasma (PPP) was stored in small portions for up to one month at −30 °C. Before each experiment, PPP was restored at 37 °C for 15 min. Once thawed, the PPP was not frozen again nor used for retesting.

### 4.7. Materials for Biological Studies

Basic coagulation parameters were determined using Bio-Ksel reagents (Grudziądz, Poland): APTT reagent, calcium chloride, Bio-Ksel PT plus reagent (tromboplastin and solvent) and thrombin (3.0 UNIH/mL) for TT experiments. Calibration of the methods and calculation of coefficient of variation (PT, APTT, TT experiments) were performed using a calibrator (Bio-Ksel, Grudziadz, Poland), normal plasma (Bio-Ksel, Grudziądz, Poland) and water for injection (Polpharma, Gdańsk, Poland).

CL-test was performed using thrombin (Biomed, Lublin, Poland) and recombinant tissue plasminogen activator (t-PA, Boehringer-Ingelheim, Ingelheim am Rhein, Germany), Tris-buffered saline (TBS, Polish Chemical Reagents, Gliwice, Poland) and sodium chloride (Polish Chemical Reagents, Gliwice, Poland).

Red blood cell lysis assay was conducted using Triton X-100 (Polish Chemical Reagents, Gliwice, Poland).

Human umbilical vein endothelial cells (HUVEC) were purchased from Lonza (Clonetics, Basel, Switzerland), and cultured according to the manufacturer’s guidelines. The reagents for HUVECs included: EGM-2—medium + bullet kit (Lonza, Basel, Clonetics, Switzerland), trypsin-EDTA—0.05% solution (Sigma, St. Louis, MO, USA), trypsin neutralizing solution (Lonza, Clonetics, Switzerland), and HEPES buffered saline solution (Lonza, Clonetics, Basel, Switzerland). Cell viability was assessed using WST-1 assay (Takara, Takara Bio Europe, Saint-Germain-en-Laye, France). Glucose uptake studies were performed using 2-NBDG (2-(N-(7-Nitrobenz-2-oxa-1,3-diazol-4-yl)Amino)-2-Deoxyglucose (Thermo Fisher Scientific, Invitrogen, Waltham, MA, USA). The generation of intracellular ROS production was conducted using 2′,7′-dichlorodihydrofluorescein diacetate (Sigma, St. Louis, MO, USA). Deficient plasma factor X (Bio-Ksel, Grudziadz, Poland), thromboplastin (Bio-Ksel, Grudziądz, Poland), and 0.9% saline were used for factor X activity. The concentration of t-PA in HUVECs supernatants was performed using a human tissue plasminogen activator ELISA kit (Abcam, Cambridge, UK).

### 4.8. Glucose Utilization Assay

HUVEC cells were seeded at a density of 20,000 cells per well in 48-well plates and cultured for 24 h under standard conditions (medium EGM-2; volume 0.2 mL). The following day, the cells were treated with tested compounds at the concentrations 0.1 and 0.3 μmol/mL dissolved in culture medium (EGM-2, Lonza, Basel, Switzerland), and cultured for another 24 h. Wells treated only with EGM-2 medium (0.2 mL) constituted controls (all other conditions were the same as in the case of examined compounds). Samples treated with D-glucose (final concentration 0.1 μmol/mL) were taken as positive control. Afterwards, the cells were washed with PBS, and cultured for 2 h in glucose-free DMEM medium (with 1% BSA), followed by 30 min-incubation with insulin (final concentration 100 nmol/L). Then, the medium was discarded, and the cells were incubated with 2-NBDG (final concentration 50 μmol/L) for another 30 min. The cells were lysed using 1% TritonX-100 solution in PBS. The fluorescence was read using a microplate reader (BioTek Instruments, Winooski, VT, USA) at 480/530 nm. 

The concentration of 2-NBDG in each well was calculated using a calibration curve that was prepared by spiking the known amount of 2-NBDG (0.1–25 μmol/L) in lysis buffer, followed by fluorescence measurement. The results are presented as the mean ± SD, *n* = 4. The coefficients of variation for the assay were determined (CV = 5.6%, *n* = 8).

### 4.9. Basic Coagulation Tests: PT, INR, APTT, TT

The effects of sulfonamide derivatives (**1**–**10**) on the basic coagulation parameters (i.e., PT, INR, APTT, TT) were conducted on a coagulometer (CoagChrom-3003 Bio-Ksel, Grudziadz, Poland) according to a routine procedure described previously [21]. The experiments were conducted in multiplicates (*n* = 5–6), and their results are presented as mean ± standard deviation (SD). Control samples consisting of distilled water and methanol (2:3) were performed.

All methods were validated using Bio-Ksel normal plasma, which was dissolved in water for injection (Polpharma, Gdańsk, Poland). Coefficients of variability for all tests were as follows, PT: W = 4.85%, INR: W = 4.72%, APTT: W = 1.91%, TT: W = 1.18%). The reference values for each test are as follows: PT: 9.7–13.8 s; INR: 0.9–1.2; APTT: 28.2–42.3 s; TT: 11.0–16.5 s for 3.0 UNIH/mL of thrombin.

### 4.10. Red Blood Cells Lysis Assay and Morphology

The influence of metformin derivatives (**1**–**10**) on RBC membrane integrity was conducted by lysis assay which was conducted as described elsewhere [22]. First, 2% RBC suspension in 0.9% saline was thoroughly vortexed, and incubated at 37 °C for one hour (static conditions, incubator, Falc Instruments, Treviglio, Italy) with the tested compounds at concentrations ranging from 0.006 to 1.5 µmol/mL or pure 0.9% NaCl (control). Afterwards, the samples were centrifuged at 1000× *g* for 10 min and the absorbance of the supernatant was recorded at 550 nm.

The results are presented as the degree of hemolysis, which constituted a percentage of the released hemoglobin. A sample containing 10 μL of 2.0% *v*/*v* Triton X-100 was used as a positive control (100% of hemolysis), whereas a sample of saline solution represented spontaneous hemolysis of RBCs (control). The results are presented as mean ± standard deviation (SD), *n* = 4. The coefficient of variability was calculated: W = 11.95%, *n* = 7.

The samples for RBCs morphology were prepared according to the same procedure. Microscopy studies were conducted using a phase contrast Opta-Tech inverted microscope, at 400-times magnification, equipped with software (OptaView 7, Warsaw, Poland) for image analysis.

### 4.11. HUVEC Cell Growth

WST-1 assay (Takara, Takara Bio Europe, Saint-Germain-en-Laye, France) was used to assess the effects of synthesized derivatives (**1**–**10**) on the growth of HUVEC cells. The experiments were conducted as described previously [20]. HUVECs were seeded on 96-well microplates at a density of 7500 and cultured for 24 h followed by treatment with compounds or pure medium (control) (v = 100 μL) for another 24 h (37 °C, 5% CO_2_). Afterwards, the cells were washed with culture medium (100 μL) and WST-1 reagent diluted in medium (10 µL of compound + 90 μL of medium) was added. The plates were incubated at 37 °C in 5% CO_2_ for another 90 min, and the absorbance was read at 450 nm using a microplate reader (iMARK, Bio-Rad, Hercules, CA, USA).

HUVECs viability is expressed as a percentage of the control samples which constituted 100% viability. The experiments were conducted in multiplicates (*n* = 8), and the results are presented as mean ± SD. The variability coefficient of the method was calculated (CV = 5.25%, *n* = 8). The influence of synthesized derivatives on morphology of HUVECs was examined using an inverted microscope with phase contrast (magnification 100×) (Opta-Tech, software OptaView 7, Warsaw, Poland).

### 4.12. Monitoring Endothelial Cell Migration in Real Time

HUVEC cells were cultured on 96-well plates and incubated until reaching 70% confluence. After culturing for 24 h, the confluent cells were wounded by scratching with a manufacturer provided scratcher (NanoEntek, Seoul, Korea), and the wells were rinsed with 100 μL of fresh medium. Afterwards, medium was replaced with the same volume of fresh medium (control) or medium including compounds at various concentrations (10 + 90 μL, final concentration of compounds 0.1 and 0.5 μmol/mL). The plates were incubated up to 36 h at 37 °C (5% CO_2_). In this period, migration of cells was monitored using the JuLI™ Stage system which is a Real-Time CHR (Cell History Recorder, NanoEntek, Seoul, Korea) designed for live cell imaging and analysis. The images of cells migration were acquired continuously every 10 min (high-sensitivity monochrome CCD (Sony sensor 2/3”, Tokyo, Japan)). The images were analyzed by dedicated software (NanoEntek, Seoul, Korea), and the width of the scratch area was measured. The results are presented as mean ± SD, *n* = 4–8. The coefficients of variation for the applied method were determined (CV = 13.6–16.0%, depending on the time point, *n* = 8)

### 4.13. Clot Formation and Lysis Test (CL-Test)

Selected compounds (**2**, **3**, **4**, **6**, **10**) were further examined for their potential to affect the process of clot formation and fibrinolysis. For this purpose, we performed CL-test, which is based on the continuous measurement of the changes in optical transmittance over time, as described by Kostka et al. [51] and Sikora et al. [52].

General conditions of the experiments were the same as the ones published previously [22,52]. The process of clot formation was induced by thrombin (10 µL, final concentration 0.5 IU/mL), and fibrinolysis was triggered by t-PA (10 µL, final concentration in a sample 220 ng/mL). The measurements were conducted on three-fold diluted pooled human citrate plasma (3H Biomedical, Uppsala, Sweden). Tested compounds in a volume of 10 µL to 470 µL of diluted plasma (final concentration 0.1–1.0 µmol/mL) were added. The clot formation and lysis curves were recorded at λ = 405 nm, by means of a spectrophotometer (Cecil CE 2021 (London, England)) with circulating thermostated water (37 °C) and a magnetic stirrer (Electronic Stirrer Model 300 Rank Brothers Ltd, Cambridge, England). The experiments were conducted in multiplicates (*n* = 5–8), and the results are presented as mean ± SD.

The obtained curves were analyzed by means of dedicated software [51], used to measure parameters of clot formation, its stabilization and fibrinolysis. Parameters of the clot formation were the following: Tt—thrombin time [s], Fmax—maximum clotting [% T], Tf—plasma clotting time [s], Fvo—initial plasma clotting velocity [% T/min], Sr—area under the clot formation curve [%Txmin]; parameters of the clot stabilization phase included: Tc—clot stabilization time [s], Sc—area under the curve of stable clot formation [% Txmin]; parameters of fibrinolysis included: Lmax—maximum lysis [% T], Tl—fibrinolysis time [s], Lvo—initial clot fibrinolysis velocity [% T/min], Sf—area under the fibrinolysis curve [% Txmin]. The overall potential of clot formation and fibrinolysis (CL_AUC_, [% Txmin]) and the total time of the process of clot formation and fibrinolysis (T, [s]) were also estimated.

The method of clot formation and fibrinolysis was validated, and the coefficient of variation (W) for pooled human plasma (*n* = 8) was within the range 3.74–14.53, depending on the calculated parameter.

### 4.14. Factor X Activity

PPP was diluted with 0.9% NaCl (1 + 4) and incubated with 50 μL of deficient plasma factor X and 10 μL of the tested compound or water (control) at 37 °C for 1 min. Afterwards, the PT reagent (thromboplastin, 100 µL) was added. PT time was recorded (coagulometer CoagChrom-3003, Bio-Ksel, Grudziadz, Poland) as the time needed for clot formation after adding the reagent. The activity of factor X was counted on the basis of a calibration curve (R^2^ = 0.999) performed on a calibrator diluted in the range 1:5–1:80 [24]. The observed change in plasma coagulation time is proportional to the concentration (% activity) of factor X in the tested plasma treated with the analyzed compounds. The coefficient of variability for the method was calculated: W = 1.80%, and the reference values for factor X activity ranged between 77 and 131%.

### 4.15. Plasminogen Activators Release from Endothelial Cells

HUVEC cells were seeded at a density of 10,000 per well on 96-well plates and incubated for 24 h. Afterwards 100 μL of medium was replaced with the same volume of fresh medium (control) or medium containing compounds (**2**, **3**, **4**, **6** or **10**) at two concentrations (0.1 and 0.3 µmol/mL). The plates were incubated at 37 °C (5% CO_2_) for next 24 h. Cell supernatants were then collected into Eppendorf tubes and stored at −20 °C until analysis. Before the analysis, the samples were thawed at room temperature for 15 min, and diluted 2.5-fold with diluent in an ELISA kit.

Measurements of t-PA concentration in HUVECs supernatant were made according to the manufacturer’s protocol. A t-PA specific antibody has been pre-coated onto 96-well plates and blocked. Standard samples (0–1 ng/mL) of t-PA or test samples in a volume of 50 μL were added and incubated for 2 h. The plate was washed five times with washing buffer (200 μL), and subsequently, a t-PA specific biotinylated detection antibody (50 µL) was added followed by washing with wash buffer. Then, streptavidin-peroxidase conjugate was added (50 µL) and unbound conjugates were washed away with wash buffer. Chromogenic substrate (TMB; 3,3′,5,5′-tetramethylbenzidine) was used to visualize enzymatic reaction (blue color product). The last step involved adding acidic stop solution and making immediately absorbance measurements at a wavelength of 450 nm (iMARK, Bio-Rad, Hercules, CA, USA). The concentration of t-PA in HUVECs supernatant was calculated using a calibration curve (R^2^ = 0.994). The results are presented as mean ± SD, *n* = 6. The coefficient of variability for the method, W = 7.21%.

### 4.16. Intracellular ROS Generation

HUVEC cells were seeded in 24-well plates at a density 50,000 cells per well and cultured for 24 h under standard conditions (volume 0.3 mL). Next, the cells were exposed to the tested compounds (**2**, **3**, **4**, **6** or **10**) at the concentrations of 0.1 and 0.3 µmol/mL or ascorbic acid as reference substance. After 24 h treatment, control and treated cells were collected (washed with PBS, and harvested with accutase), and transferred to Eppendorf tubes. The cells were centrifuged (5 min, 220× *g*). Then, the supernatant was discarded, cold staining buffer (Biolegend, London, UK) was added (0.5 mL), and the cells were centrifuged (200× *g*, 5 min.). The solution was discarded, and the cells were resuspended in 200 µL of cell staining buffer. Then, 2 µL of H_2_DCFDA (2′,7′-dichlorodihydrofluorescein diacetate) (final concentration 2 µmol/L) was added, mixed and incubated for 30 min in the dark at room temperature. The final volume of sample was 250 µL. The fluorescence was measured by flow cytometry (CytoFlex, blue laser, 480 nm, Beckman-Coulter, Indianapolis, IN, US). The results were analyzed using Kaluza 2.1 (Beckman-Coulter Inc., Brea, CA, USA) software. In total, 10,000 cells were analyzed from each sample and the data were presented as the mean ± SD of three independent experiments. The coefficients of variation for the assay was determined (CV = 8.26%, *n* = 3).

### 4.17. Statistical Analysis

Statistical analysis was conducted using commercially-available packages (Statistica 12.0, StatSoft Polska, Kraków, Poland; GraphPad Prism 5, San Diego, CA, USA). The normality of the distribution of continuous variables was verified with the Shapiro-Wilk test, while the homogeneity of variances was checked using the Levene test. The paired *t*-test was used to test the dependent variables (e.g., studies on biological material), while statistically significant differences between the means of independent groups were identified using a one-way ANOVA. The variables with non-normal distributions were compared using the Wilcoxon signed rank test. The results of all the tests were considered significant at *p*-values lower than 0.05.

## 5. Conclusions

Our novel findings show that sulfonamides with two or three methyl groups in the aromatic ring (**6**, **7**, **9**, **10**), similarly to the parent drug, metformin, significantly increase glucose uptake in HUVECs. Importantly, all examined compounds were found to significantly prolong both APTT and TT, which proves their anti-coagulant properties. Furthermore, compounds **4** and **6** affected all measured coagulation parameters, including PT, APTT and TT. These results were further confirmed in Xa activity assay in which its decreased activity was reported in the presence of tested compounds. Furthermore, in-depth studies using multiparametric CL-test confirmed that the compounds slow the process of fibrin polymerization, and increase the time it takes to start the clot formation process. Importantly, none of the tested compounds increases the overall potential of clot formation and fibrinolysis (CL_AUC_). Results of previous studies [22] demonstrated that metformin affected neither the intrinsic and extrinsic coagulation pathway (APTT, PT) nor the kinetic parameters of clot formation and fibrinolysis (CL-test). However, our current results suggest that benzenesulfonamides with methyl substituents in the aromatic ring exert advantageous anti-coagulant activity. Essentially, erythrotoxicity studies showed that none of the synthesized compounds exert an adverse effect on erythrocyte integrity, and do not contribute to pathological changes in the shape of cells, which means that the compounds do not interact strongly with the lipid-protein bilayer.

In conclusion, chemical transformation of metformin backbone into sulfonamides with alkyl substituents in the aromatic ring leads to the formation of potential dual-action agents with comparable glucose-lowering properties and stronger anti-coagulant activity than those of the parent drug, metformin. Therefore, the outcomes of this study can be considered an initial encouraging step in the development of novel biguanide-based compounds possessing both anti-hyperglycemic and anti-coagulant properties.

## Figures and Tables

**Table 1:** Results of stability studies of synthesized compounds **1**–**10** (at the concentrations of 100 µmol/L) after 6 h-incubation (37 °C) in Tris buffer (pH = 7.4), human plasma and rat liver S9 fraction. The results are presented as mean ± SD, *n* = 3.

Compound	Tris Buffer [%]	Human Plasma [%]	Rat Liver S9 Fraction [%]
**1**	99.7 ± 1.3	105.1 ± 1.9	104.6 ± 1.4
**2**	91.3 ± 12.8	121.4 ± 4.0	124.4 ± 9.0
**3**	95.7 ± 2.5	112.1 ± 6.6	131.5 ± 15.5
**4**	100.7 ± 2.5	106.3 ± 2.3	106.1 ± 2.7
**5**	94.9 ± 5.2	109.5 ± 1.9	107.7 ± 0.7
**6**	131.7 ± 7.9	99.8 ± 8.9	91.4 ± 4.0
**7**	102.3 ± 4.8	96.4 ± 5.9	100.6 ± 5.1
**8**	105.8 ± 3.8	114.5 ± 3.4	110.6 ± 12.9
**9**	106.4 ± 12.3	102.3 ± 3.8	93.2 ± 2.3
**10**	39.4 ± 8.4	103.4 ± 2.9	105.5 ± 9.6

**Table 2:** Effects of synthesized compounds **1**–**10** on basic coagulation parameters.

Compound	Concentration [µmol/mL]	APTT [s]	PT [s]	INR	TT [s]
**1**	0.0 (CTR)	37.4 ± 7.2	13.2 ± 1.1	1.01 ± 0.1	14.1 ± 1.1
	0.006	38.3 ± 7.5	13.4 ± 0.9	1.1 ± 0.1	15.4 ± 1.8
	0.06	37.4 ± 7.6	12.9 ± 0.7	1.0 ± 0.1	16.7 ± 2.1
	0.3	37.6 ± 7.4	12.9 ± 1.0	1.0 ± 0.1	16.1 ± 2.9
	0.6	38.4 ± 7.4	13.4 ± 1.0	1.1 ± 0.1	15.8 ± 2.1
	1.0	39.1 ± 4.4	13.2 ± 1.0	1.1 ± 0.1	15.1 ± 2.0
**2**	0.0 (CTR)	39.0 ± 1.4	13.5 ± 1.1	1.1 ± 0.1	12.8 ± 0.9
	0.006	39.0 ± 1.3	13.2 ± 1.3	1.1 ± 0.1	13.3 ± 1.0
	0.06	39.4 ± 0.6	13.1 ± 1.0	1.1 ± 0.1	13.0 ± 1.0
	0.3	39.2 ± 1.1	13.6 ± 1.0	1.1 ± 0.1	14.4 ± 1.2
	0.6	38.4 ± 0.5	13.3 ± 1.1	1.1 ± 0.1	**15.4 ± 1.0**
	1.0	**46.2 ± 1.5**	13.5 ± 0.9	1.1 ± 0.1	**16.3 ± 1.6**
**3**	0.0 (CTR)	40.0 ± 1.7	13.8 ± 0.7	1.1 ± 0.1	13.6 ± 0.3
	0.006	39.5 ± 1.0	13.6 ± 0.9	1.1 ± 0.1	13.8 ± 1.2
	0.06	35.5 ± 1.4	13.0 ± 0.8	1.0 ± 0.1	13.6 ± 0.5
	0.3	37.4 ± 1.3	13.2 ± 1.1	1.0 ± 0.1	15.2 ± 0.5
	0.6	**41.3 ± 3.6**	13.1 ± 1.3	1.0 ± 0.1	**18.1 ± 0.9**
	1.0	**47.7 ± 1.9**	13.6 ± 1.0	1.1 ± 0.1	**21.7 ± 1.2**
**4**	0.0 (CTR)	33.2 ± 1.9	12.5 ± 0.5	1.0 ± 0.1	12.9 ± 1.7
	0.006	33.7 ± 4.1	12.7 ± 0.7	1.0 ± 0.1	12.9 ± 1.6
	0.06	32.2 ± 2.2	12.4 ± 0.6	1.0 ± 0.1	13.2 ± 1.2
	0.3	34.5 ± 3.1	12.3 ± 0.6	1.0 ± 0.1	14.8 ± 1.6
	0.6	35.4 ± 3.4	12.7 ± 0.7	1.0 ± 0.1	**16.4 ± 1.6**
	1.0	**47.2 ± 3.3**	**14.1 ± 1.8**	**1.1 ± 0.1**	**19.5 ± 0.4**
**5**	0.0 (CTR)	33.1 ± 2.5	13.0 ± 1.0	1.1 ± 0.1	12.3 ± 0.8
	0.006	31.6 ± 1.7	12.6 ± 0.8	1.0 ± 0.1	12.4 ± 1.0
	0.06	33.4 ± 2.4	12.5 ± 0.7	1.0 ± 0.1	13.5 ± 2.1
	0.3	33.2 ± 3.3	12.6 ± 0.9	1.0 ± 0.1	14.7 ± 1.7
	0.6	37.8 ± 1.8	12.8 ± 1.0	1.0 ± 0.1	**17.2 ± 3.7**
	1.0	**45.1 ± 5.4**	13.5 ± 1.3	1.1 ± 0.1	**21.1 ± 2.8**
**6**	0.0 (CTR)	38.0 ± 4.8	11.9 ± 0.9	0.9 ± 0.1	14.5 ± 1.5
	0.006	36.2 ± 4.9	11.9 ± 0.8	0.9 ± 0.1	15.1 ± 2.1
	0.06	37.2 ± 4.1	11.8 ± 0.9	0.9 ± 0.1	15.5 ± 2.1
	0.3	39.3 ± 5.3	12.7 ± 1.2	1.0 ± 0.1	17.9 ± 2.6
	0.6	48.8 ± 6.0	12.5 ± 0.9	1.0 ± 0.1	**22.4 ± 2.8**
	1.0	**53.6 ± 7.0**	**13.1 ± 0.6**	**1.1 ± 0.0**	**31.1 ± 3.2**
**7**	0.0 (CTR)	37.7 ± 3.2	12.1 ± 1.0	1.0 ± 0.1	14.5 ± 1.9
	0.006	37.7 ± 2.8	11.9 ± 0.6	0.9 ± 0.1	14.5 ± 1.3
	0.06	36.6 ± 2.3	12.1 ± 0.5	1.0 ± 0.0	15.5 ± 1.8
	0.3	39.2 ± 4.2	12.3 ± 0.4	1.0 ± 0.0	17.5 ± 2.8
	0.6	44.3 ± 5.7	12.1 ± 0.4	1.0 ± 0.0	**21.4 ± 3.4**
	1.0	**49.8 ± 4.8**	12.3 ± 0.5	1.0 ± 0.0	**25.7 ± 4.4**
**8**	0.0 (CTR)	38.3 ± 4.7	12.9 ± 1.2	1.0 ± 0.1	13.4 ± 1.2
	0.006	38.8 ± 6.3	13.4 ± 1.7	1.1 ± 0.1	12.8 ± 1.5
	0.06	38.2 ± 4.9	13.2 ± 1.2	1.1 ± 0.1	13.5 ± 2.4
	0.3	39.8 ± 5.4	13.5 ± 1.1	1.1 ± 0.1	14.6 ± 2.9
	0.6	43.6 ± 5.3	13.5 ± 1.3	1.1 ± 0.1	16.9 ± 2.6
	1.0	**48.3 ± 5.3**	14.0 ± 1.2	1.1 ± 0.1	**20.1 ± 4.0**
**9**	0.0 (CTR)	37.8 ± 3.4	13.6 ± 0.9	1.1 ± 0.1	13.8 ± 1.2
	0.006	38.9 ± 4.4	13.2 ± 0.8	1.1 ± 0.1	13.3 ± 1.2
	0.06	38.5 ± 4.0	13.2 ± 0.7	1.1 ± 0.1	13.9 ± 1.1
	0.3	39.9 ± 4.0	13.2 ± 1.0	1.1 ± 0.1	15.3 ± 1.6
	0.6	42.5 ± 5.4	13.6 ± 1.2	1.1 ± 0.1	**16.7 ± 1.0**
	1.0	**44.8 ± 5.0**	13.8 ± 1.2	1.1 ± 0.1	**18.0 ± 1.0**
**10**	0.0 (CTR)	39.1 ± 4.3	12.9 ± 0.9	1.0 ± 0.1	15.1 ± 2.0
	0.006	39.4 ± 4.0	13.2 ± 1.1	1.1 ± 0.1	15.3 ± 2.1
	0.06	39.5 ± 4.5	13.1 ± 1.2	1.0 ± 0.1	15.1 ± 1.7
	0.3	41.2 ± 4.5	13.2 ± 1.1	1.0 ± 0.1	18.0 ± 2.8
	0.6	**49.6 ± 7.5**	13.2 ± 1.0	1.0 ± 0.1	**21.8 ± 2.6**
	1.0	**52.6 ± 6.0**	13.8 ± 1.1	1.1 ± 0.1	**31.0 ± 4.0**
MET^#^	0.0 (CTR)	30.3 ± 4.9	12.3 ± 0.7	1.0 ± 0.1	14.9 ± 1.4
	0.06	29.7 ± 2.8	12.4 ± 0.2	1.0 ± 0.0	15.1 ± 1.0
	0.3	31.0 ± 3.0	12.5 ± 1.1	1.0 ± 0.1	15.2 ± 1.3
	0.6	31.2 ± 2.0	13.0 ± 1.2	1.1 ± 0.1	15. 4 ± 1.3
	1.2	31.9 ± 2.92	13.1 ± 1.6	1.2 ± 0.1	15.4 ± 1.3

Abbreviations: APTT—Activated Partial Thromboplastin Time; PT—Prothrombin Time, TT—Thrombin Time; INR—International Normalized Ratio. The results are presented as mean ± standard deviation, *n* = 4–6. Values in bold are statistically significant in comparison to control samples, *p* < 0.05. The results for metformin, marked with a hashtag, were published previously [20]. Metformin was not found to affect any measured parameter. The reference values: PT: 9.7–13.8 s; INR: 0.9–1.2; APTT: 28.2–42.3 s; TT: 11.0–16.5 s for 3.0 UNIH/mL of thrombin (international unit).

**Table 3:** The effects of sulfonamides on HUVEC cells growth. The results (viability, %) are presented as mean ± SD (*n* = 6–8).

Compound	Control	0.006 µmol/mL	0.06 µmol/mL	0.3 µmol/mL	0.6 µmol/mL	1.5 µmol/mL
**1**	100.0 ± 3.7	92.1 ± 9.6	89.9 ± 3.0	85.8 ± 5.4	62.0 ± 3.5	46.8 ± 1.8
**2**	100.0 ± 5.1	99.4 ± 7.4	95.1 ± 5.3	70.2 ± 3.4	27.1 ± 3.8	12.3 ± 3.0
**3**	100.0 ± 5.1	97.7 ± 3.9	91.4 ± 3.7	78.6 ± 4.9	22.1 ± 4.8	11.1 ± 2.9
**4**	100.0 ± 3.6	94.7 ± 5.6	91.8 ± 6.6	89.5 ± 7.2	67.6 ± 7.6	40.0 ± 8.6
**5**	100.0 ± 3.7	92.4 ± 8.0	92.7 ± 8.9	74.3 ± 3.6	66.9 ± 20.1	47.7 ± 7.3
**6**	100.0 ± 5.1	99.3 ± 7.1	96.4 ± 2.5	78.1 ± 8.8	45.0 ± 5.1	32.9 ± 3.6
**7**	100.0 ± 4.7	82.2 ± 5.2	77.9 ± 7.2	55.3 ± 5.0	26.5 ± 6.3	7.1 ± 1.4
**8**	100.0 ± 6.0	90.1 ± 6.9	83.8 ± 5.3	62.1 ± 5.3	22.4 ± 4.0	2.5 ± 0.8
**9**	100.0 ± 4.7	83.9 ± 8.5	70.9 ± 4.4	56.1 ± 6.5	24.9 ± 6.6	5.6 ± 1.2
**10**	100.0 ± 4.7	93.2 ± 5.3	87.9 ± 4.4	60.1 ± 5.4	19.6 ± 5.2	3.4 ± 1.4
Metformin ^#^	100.03 ± 8.9	103.4 ± 9.4	104.6 ± 5.6	106.2 ± 5.2	104.6 ± 6.7	102.0 ± 4.6

^#^ The results of the effects of metformin on HUVEC viability was published in our previous paper [23].

**Table 4:** The effects of selected sulfonamides on the release of t-PA from HUVEC cells. The results (t-PA concentration in supernatants, pg/mL) are presented as mean ± SD (*n* = 6). Values in bold denote a significant difference between tested compounds and control samples (*p* < 0.05).

Compound	Concentration [µmol/mL]	Released t-PA [pg/mL]
**CTR**	-	2883.6 ± 138.7
**2**	0.1	**1553.5 ± 305.2**
	0.3	**544.5 ± 80.9**
**3**	0.1	**2170.8 ± 215.6**
	0.3	**1137.8 ± 149.2**
**4**	0.1	**1566.6 ± 137.3**
	0.3	**845.1 ± 31.6**
**6**	0.1	**1422.7 ± 219.2**
	0.3	**1113.3 ± 407.3**
**10**	0.1	**1060.8 ± 190.9**
	0.3	**484.2 ± 100.9**

## Supplementary Materials

The following are available online at https://www.mdpi.com/1424-8247/13/10/323/s1, Table S1. The effects of compounds **1**–**10** on the migration of human endothelial cells (HUVECs) analyzed in the JuliStage system. The results are presented as a wound width (µm). Figure S1. Effects of metformin and synthesized compounds **1**–**10** on the 2-NBDG uptake in HUVEC cells. Figure S2. Effect of metformin at various concentrations on erythrocytes morphology. Figure S3. Effect of synthesized compounds **1**–**10** on endothelial cell (HUVECs) viability and morphology after 24-h incubation. Figure S4. Inhibition of cell migration in the presence of metformin and compound **10**. HUVEC cells migration was evaluated using wound healing assay in the JuLiStage system. Table S2. The effects of compound **2** on the kinetic parameters of clot formation and fibrinolysis. Table S3. The effects of compound **3** on the kinetic parameters of clot formation and fibrinolysis. Table S4. The effects of compound **4** on the kinetic parameters of clot formation and fibrinolysis. Table S5. The effects of compound **6** on the kinetic parameters of clot formation and fibrinolysis. Table S6. The effects of compound **10** on the kinetic parameters of clot formation and fibrinolysis.
