# Supplementary material for: Novel Sulfonamide-Based Analogs of Metformin Exert Promising Anti-Coagulant Effects without Compromising Glucose-Lowering Activity
Source: Pharmaceuticals (Basel). 2020 Oct 21;13(10):323. doi: 10.3390/ph13100323 (PMC7589829; doi:10.3390/ph13100323)
Supplement: Supplementary file 1 [file pharmaceuticals-13-00323-s001.pdf]

SUPPORTING MATERIALS:

NOVEL SULFONAMIDE-BASED ANALOGS OF METFORMIN EXERT PROMISING ANTI-COAGULANT EFFECTS WITHOUT COMPROMISING GLUCOSE-LOWERING ACTIVITY

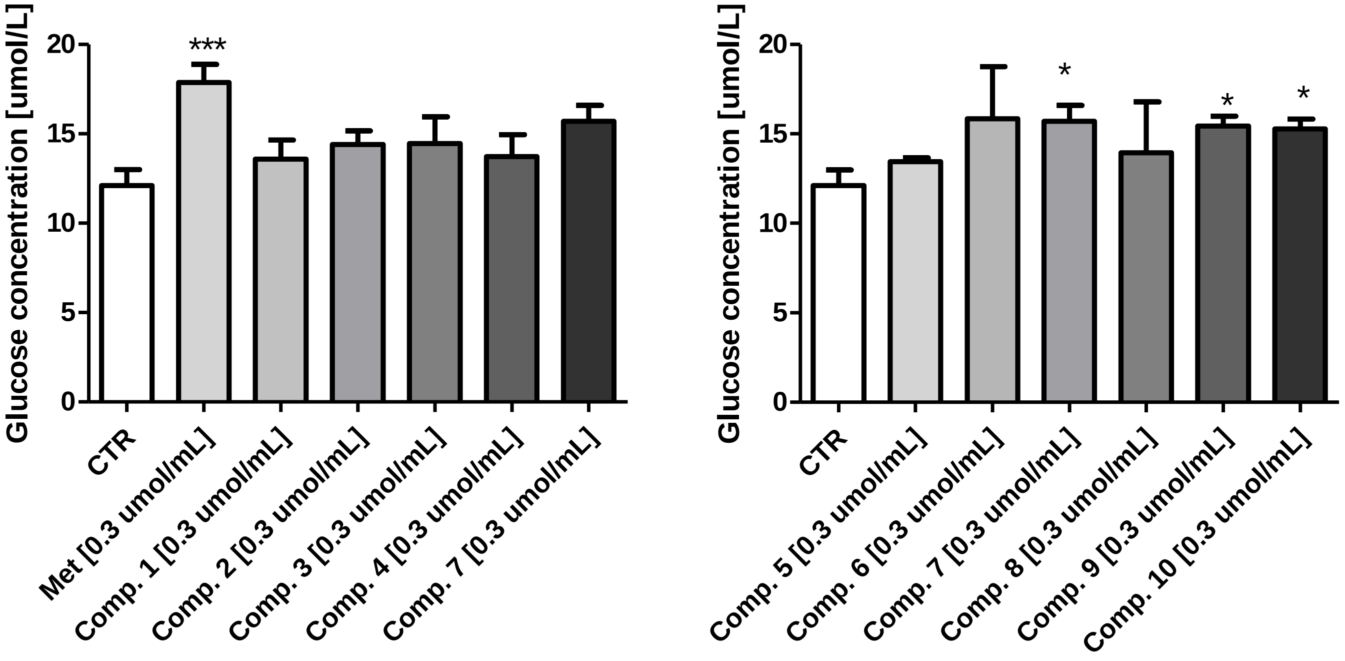

Figure S1. *Effects of metformin and synthesized compounds 1 – 10on the 2-NBDG uptake in HUVEC cells. The cells were treated with tested compounds at 0.3 µmol/mL for 24 hours prior to the experiments. On the day of the experiment the cells were glucose-starved for two hours, then incubated with insulin (30 minutes), and in sequence with 2-NBDG for 45 minutes. The results are presented as mean ± SD, n = 4 – 6. Significant differences in glucose uptake are denoted with asterisk; * p < 0.05, ** p < 0.01, *** p < 0.001.*

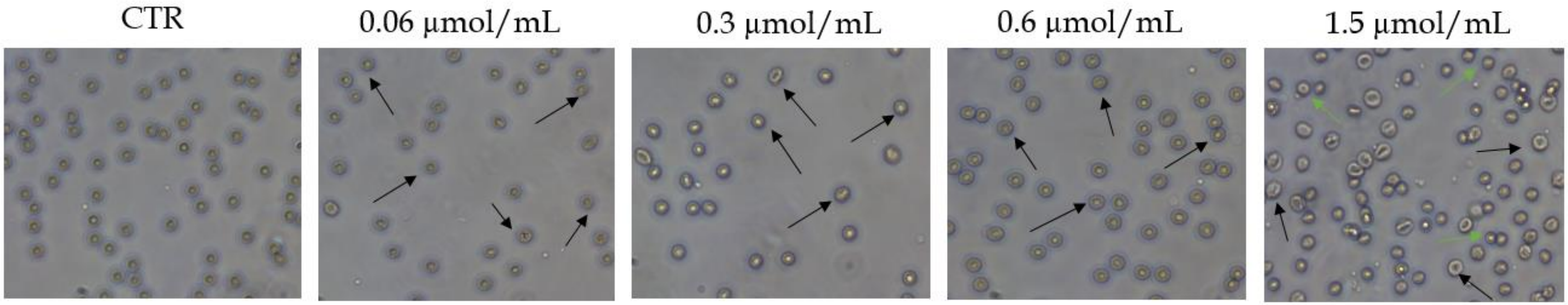

Figure S2. *Effect of metformin at various concentrations on erythrocytes morphology. Representative phase-contrast images are shown (magnification of 400 times), echinocytes are marked with black arrows, eryptotic RBCs are marked with green arrows.*

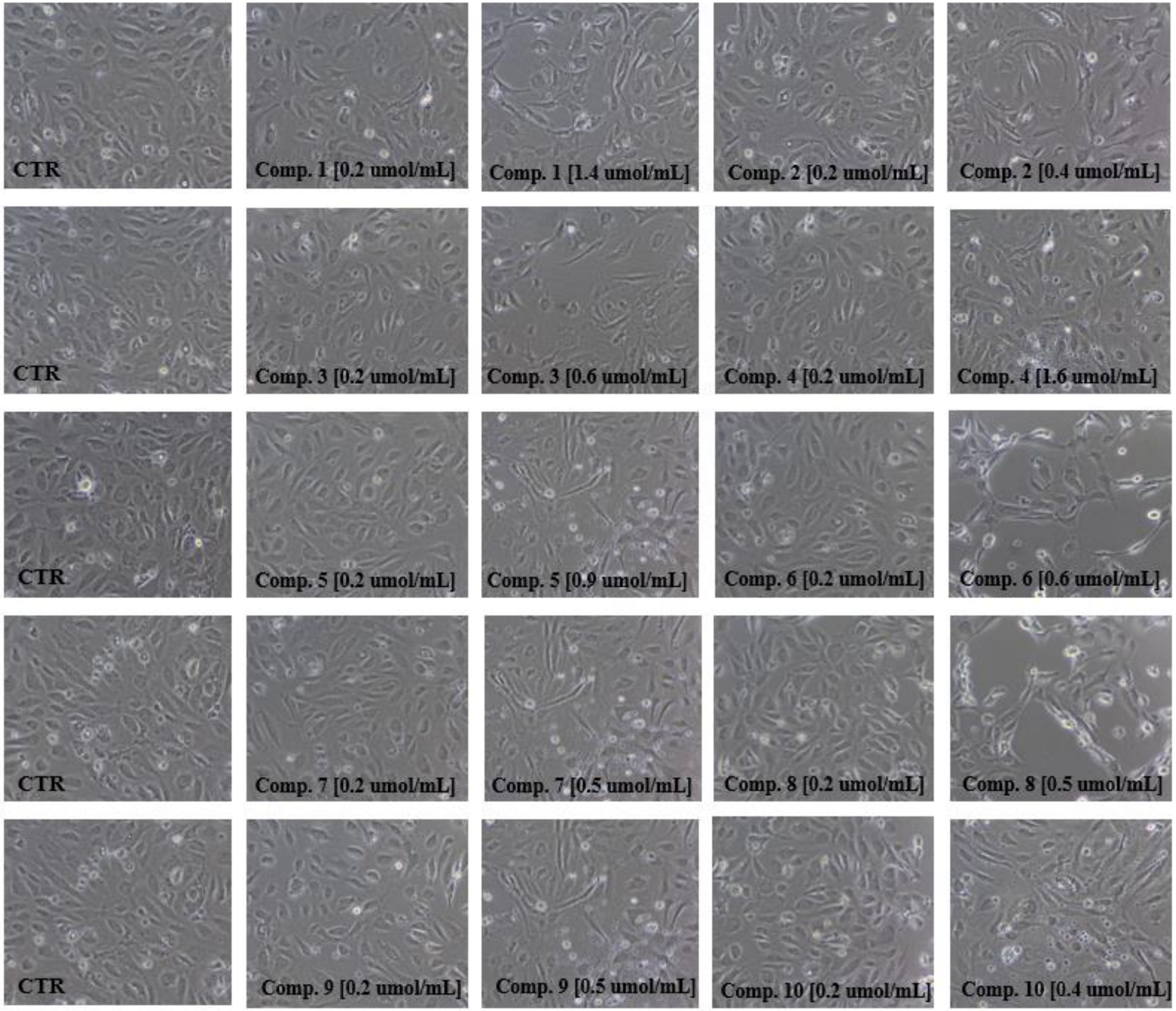

Figure S3. *Effect of synthesized compounds* ***1*****–*****10*** *on endothelial cell (HUVECs) viability and morphology after 24-h incubation. HUVECs were cultured without (control, CTR) and in the presence of compounds* ***1*****–*****10*** *at a concentration of 0.010 – 1.5 μmol/mL. Representative cell images are shown for concentration of 0.2 μmol/mL and concentration equaling* IC_{50} *value of each compound (100-fold magnification).*

Table S1. *The effects of compounds **1 - 10** on the migration of human endothelial cells (HUVECs) analyzed in the JulieStage system. The results are presented as a wound width [µm].*

Compound	Concentration	Start [µm]	6 hours [µm]	12 hours [µm]	18 hours [µm]	24 hours [µm]
**CTR**	-	690.32 ± 159.01	575.25 ± 121.88	462.15 ± 103.78	362.33 ± 101.44	320.70 ± 105.76
**1**	0.1 µmol/mL	595.11 ± 24.91	488.04 ± 77.15	364.40 ± 86.87	260.15 ± 62.48	259.03 ± 44.90
	0.5 µmol/mL	628.28 ± 129.75	533.43 ± 118.64	499.30 ± 112.08	470.80 ± 102.56	468.58 ± 113.26
**2**	0.1 µmol/mL	580.05 ± 188.74	439.51 ± 52.76	358.11 ± 26.21	288.81 ± 27.51	266.68 ± 31.53
	0.5 µmol/mL	599.33 ± 51.31	545.40 ± 41.38	494.38 ± 102.42	495.28 ± 110.55	515.87 ± 75.12
**3**	0.1 µmol/mL	607.16 ± 51.15	471.20 ± 43.97	331.43 ± 56.69	255.82 ± 35.88	249.57 ± 22.27
	0.5 µmol/mL	597.51 ± 46.52	526.48 ± 54.07	441.75 ± 67.73	410.36 ± 83.32	437.84 ± 110.16
**4**	0.1 µmol/mL	676.18 ± 131.51	506.54 ± 190.07	426.31 ± 136.17	339.30 ± 138.65	271.63 ± 85.17
	0.5 µmol/mL	667.43 ± 145.83	569.57 ± 123.67	496.38 ± 141.56	404.06 ± 140.61	387.53 ± 157.68
**5**	0.1 µmol/mL	628.00 ± 73.84	516.65 ± 71.03	399.11 ± 86.53	342.76 ± 78.40	280.35 ± 94.43
	0.5 µmol/mL	769.78 ± 164.34	665.29 ± 98.75	626.94 ± 91.36	582.96 ± 135.84	555.24 ± 182.10
**6**	0.1 µmol/mL	583.67 ± 64.76	454.23 ± 42.32	385.42 ± 23.71	328.32 ± 25.01	276.10 ± 49.30
	0.5 µmol/mL	613.99 ± 126.90	536.46 ± 80.00	509.91 ± 33.73	482.51 ± 27.75	504.46 ± 45.40*
**7**	0.1 µmol/mL	592.64 ± 136.83	511.29 ± 113.13	380.65 ± 105.20	331.76 ± 77.09	288.57 ± 72.09
	0.5 µmol/mL	636.55 ± 99.88	526.35 ± 14.62	480.17 ± 57.05	453.55 ± 100.72	466.96 ± 96.94
**8**	0.1 µmol/mL	581.58 ± 48.09	462.01 ± 43.38	395.83 ± 41.14	332.64 ± 48.24	321.73 ± 39.15
	0.5 µmol/mL	593.77 ± 84.47	480.24 ± 93.26	474.54 ± 103.25	453.68 ± 129.64	464.61 ± 90.86
**9**	0.1 µmol/mL	575.79 ± 77.88	454.77 ± 70.04	407.92 ± 45.97	323.95 ± 62.11	297.58 ± 82.17
	0.5 µmol/mL	612.60 ± 25.98	485.99 ± 54.31	461.82 ± 39.85	440.81 ± 63.95	433.35 ± 83.70
**10**	0.1 µmol/mL	667.60 ± 95.11	495.40 ± 133.38	365.46 ± 59.77	297.38 ± 38.56	272.57 ± 48.23
	0.5 µmol/mL	683.83 ± 153.80	567.11 ± 96.96	536.80 ± 139.55	509.36 ± 110.74	505.06 ± 101.69*
**MET**	0.1 µmol/mL	735.26 ± 68.50	574.28 ± 127.37	457.78 ± 95.52	405.49 ± 119.08	409.97 ± 121.57
	0.5 µmol/mL	675.45 ± 37.67	553.92 ± 23.99	493.19 ± 15.74	487.72 ± 9.60	424.89 ± 36.84*

The results are presented as mean ± SD, n = 4 – 8; (* $p < 0.05$).

Figure S4. *Inhibition of cell migration in the presence of metformin and compound 10. HUVEC cells migration was evaluated using wound healing assay in JuLiStage system. Representative cell images are shown for control samples, metformin and compound 10 at the concentration of 0.5 μmol/mL. Cells were photographed at the indicated times (t = 0, 6, 12, 18, 24 h); 40× magnification.*

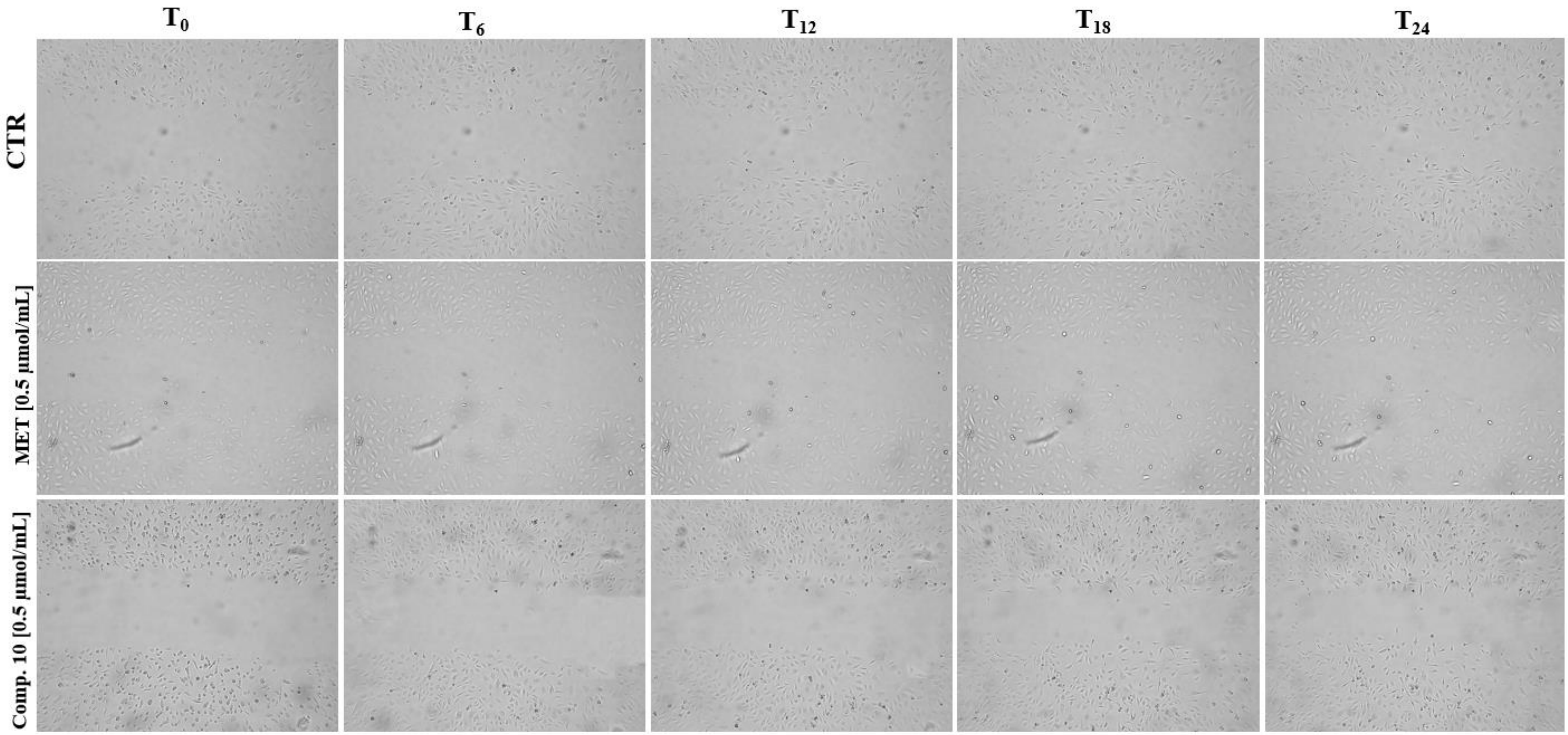

Table S2. *The effects of compound* ***2*** *on the kinetic parameters of clot formation and fibrinolysis (CL test). The results are presented as mean ±SD, n = 5 – 6. The values in bold denotes statistically significant difference versus control (p < 0.05).*

**CL-test**	**Parameter**	**Control**	**Compound 2**		
			**0.1 µmol/mL**	**0.5 µmol/mL**	**1.0 µmol/mL**
Clot formation phase	**Tt [s]**	32.70 ± 5.47	32.87 ± 3.07	33.16 ± 4.13	37.84 ± 7.63
	**Fmax [%T]**	44.60 ± 4.34	43.02 ± 5.28	39.71 ± 5.18	**38.56 ± 3.38**
	**Tf [s]**	89.89 ± 8.61	**77.22 ± 5.94**	**76.96 ± 8.50**	83.43 ± 8.31
	**Fvo [%T/min]**	64.87 ± 8.32	75.92 ± 10.05	74.74 ± 15.66	63.74 ± 10.92
	**Sr [%T·min]**	45.07 ± 7.95	37.24 ± 5.50	**34.67 ± 5.34**	**36.71 ± 6.06**
Clot stabilization phase	**Tc [s]**	225.00 ± 41.82	229.20 ± 40.08	244.50 ± 35.07	**293.67 ± 65.89**
	**Sc [%T·min]**	178.26 ± 50.64	160.08 ± 40.19	169.39 ± 41.85	182.50 ± 48.38
Fibrinolysis phase	**Lmax [%T]**	45.07 ± 3.04	43.47 ± 5.18	**40.31 ± 4.96**	**40.11 ± 3.14**
	**Tl [s]**	157.43 ± 24.13	157.33 ± 14.01	168.43 ± 16.70	**211.71 ± 34.57**
	**Lvo [%T/min]**	27.33 ± 4.79	21.80 ± 4.12	**19.65 ± 3.29**	**20.27 ± 2.85**
	**Sf [%T·min]**	63.67 ± 17.80	56.46 ± 9.04	52.69 ± 12.43	56.63 ± 13.40
Overal potential of clot formation and fibrinolysis	**T [s]**	501.98 ± 66.65	491.96 ± 47.03	519.55 ± 38.92	**624.18 ± 104.64**
	**CL_{AUC} [%T·min]**	287.00 ± 69.13	253.78 ± 51.89	256.74 ± 53.08	275.85 ± 65.85

Tt – thrombin time, Fmax – maximum clotting, Tf – plasma clotting time, Fvo – initial plasma clotting velocity, Sr – area under the clot formation curve; Tc – clot stabilization time, Sc – area under the curve of stable clot formation; Lmax – maximum lysis, Tl – fibrinolysis time, Lvo – initial clot fibrinolysis velocity, Sf – area under the fibrinolysis curve, CL_{AUC} - overall potential of clot formation and fibrinolysis, T - total time of the process of clot formation and fibrinolysis.

Table S3. *The effects of compound **3** on the kinetic parameters of clot formation and fibrinolysis (CL test). The results are presented as mean ±SD, n = 5 – 6. The values in bold denotes statistically significant difference versus control (p < 0.05).*

**CL-test**	**Parameter**	**Control**	**Compound 3**		
			**0.1 µmol/mL**	**0.5 µmol/mL**	**1.0 µmol/mL**
Clot formation phase	**Tt [s]**	30.62 ± 5.81	30.93 ± 2.52	36.15 ± 9.12	**41.18 ± 7.78**
	**Fmax [%T]**	45.07 ± 19.92	45.03 ± 3.57	42.73 ± 2.83	43.63 ± 3.29
	**Tf [s]**	84.70 ± 11.59	78.13 ± 2.79	84.43 ± 16.90	97.93 ± 18.76
	**Fvo [%T/min]**	73.97 ± 3.78	80.08 ± 19.20	72.05 ± 13.66	61.60 ± 9.02
	**Sr [%T·min]**	42.79 ± 6.92	39.42 ± 4.06	40.59 ± 9.16	45.92 ± 7.30
Clot stabilization phase	**Tc [s]**	166.17 ± 27.35	187.50 ± 42.54	191.50 ± 44.12	**229.00 ± 34.77**
	**Sc [%T·min]**	123.18 ± 21.88	137.74 ± 38.27	132.22 ± 31.63	**160.42 ± 16.08**
Fibrinolysis phase	**Lmax [%T]**	45.47 ± 2.58	45.75 ± 2.65	43.90 ± 2.80	43.33 ± 3.03
	**Tl [s]**	116.83 ± 10.55	130.00 ± 17.83	117.00 ± 10.03	**152.00 ± 18.92**
	**Lvo [%T/min]**	25.55 ± 4.05	25.03 ± 1.88	26.75 ± 2.70	22.98 ± 2.07
	**Sf [%T·min]**	44.86 ± 3.15	48.37 ± 10.21	42.19 ± 8.08	58.24 ± 8.92
Overal potential of clot formation and fibrinolysis	**T [s]**	398.32 ± 46.82	426.55 ± 55.72	429.08 ± 77.04	**518.93 ± 62.74**
	**CL_{AUC} [%T·min]**	207.98 ± 28.46	225.52 ± 50.51	215.00 ± 47.51	265.63 ± 25.71

Tt – thrombin time, Fmax – maximum clotting, Tf – plasma clotting time, Fvo – initial plasma clotting velocity, Sr – area under the clot formation curve; Tc – clot stabilization time, Sc – area under the curve of stable clot formation; Lmax – maximum lysis, Tl – fibrinolysis time, Lvo – initial clot fibrinolysis velocity, Sf – area under the fibrinolysis curve, CL_{AUC} - overall potential of clot formation and fibrinolysis, T - total time of the process of clot formation and fibrinolysis.

Table S4. *The effects of compound* ***4*** *on the kinetic parameters of clot formation and fibrinolysis (CL test). The results are presented as mean ±SD, n = 5 – 6. The values in bold denotes statistically significant difference versus control (p < 0.05).*

CL-test	Parameter	Control	Compound 4		
			**0.1 µmol/mL**	**0.5 µmol/mL**	**1.0 µmol/mL**
Clot formation phase	**Tt [s]**	33.08 ± 5.72	27.80 ± 5.60	**37.72 ± 5.74**	39.40 ± 7.90
	**Fmax [%T]**	45.62 ± 2.18	**47.96 ± 2.39**	46.20 ± 2.92	45.92 ± 2.49
	**Tf [s]**	86.40 ± 8.70	81.52 ± 11.41	83.22 ± 17.13	92.10 ± 14.28
	**Fvo [%T/min]**	73.44 ± 8.40	88.32 ± 23.33	86.60 ± 16.69	76.32 ± 10.45
	**Sr [%T·min]**	44.30 ± 2.53	43.48 ± 3.60	43.06 ± 9.43	45.13 ± 2.04
Clot stabilization phase	**Tc [s]**	237.60 ± 38.45	225.60 ± 65.26	231.40 ± 72.04	256.80 ± 64.89
	**Sc [%T·min]**	174.85 ± 25.61	173.93 ± 45.00	171.93 ± 51.38	189.75 ± 41.76
Fibrinolysis phase	**Lmax [%T]**	45.86 ± 2.26	**48.34 ± 3.16**	45.08 ± 3.55	46.26 ± 2.61
	**Tl [s]**	140.60 ± 8.73	144.00 ± 17.13	137.20 ± 22.97	154.80 ± 10.99
	**Lvo [%T/min]**	23.92 ± 1.31	23.88 ± 3.52	24.62 ± 4.62	23.34 ± 1.58
	**Sf [%T·min]**	55.80 ± 9.30	59.12 ± 7.04	64.19 ± 14.13	57.04 ± 7.09
Overal potential of clot formation and fibrinolysis	**T [s]**	497.68 ± 51.27	478.92 ± 81.45	489.54 ± 111.52	543.10 ± 91.27
	**CL_{AUC} [%T·min]**	274.94 ± 33.32	276.66 ± 50.70	280.62 ± 72.47	280.62 ± 72.47

Tt – thrombin time, Fmax – maximum clotting, Tf – plasma clotting time, Fvo – initial plasma clotting velocity, Sr – area under the clot formation curve; Tc – clot stabilization time, Sc – area under the curve of stable clot formation; Lmax – maximum lysis, Tl – fibrinolysis time, Lvo – initial clot fibrinolysis velocity, Sf – area under the fibrinolysis curve, CL_{AUC} - overall potential of clot formation and fibrinolysis, T - total time of the process of clot formation and fibrinolysis.

Table S5. *The effects of compound **6** on the kinetic parameters of clot formation and fibrinolysis (CL test). The results are presented as mean ±SD, n = 5. The values in bold denotes statistically significant difference versus control (p < 0.05).*

CL-test	Parameter	Control	Compound 6		
			0.1 µmol/mL	0.5 µmol/mL	1.0 µmol/mL
Clot formation phase	**Tt [s]**	35.74 ± 5.77	32.64 ± 4.50	**32.90 ± 4.87**	35.46 ± 8.79
	**Fmax [%T]**	39.08 ± 3.93	44.58 ± 1.79	42.12 ± 5.86	42.58 ± 7.70
	**Tf [s]**	79.54 ± 2.97	77.04 ± 8.60	75.66 ± 8.82	88.28 ± 17.14
	**Fvo [%T/min]**	59.98 ± 9.19	76.22 ± 17.17	77.22 ± 18.35	68.40 ± 26.40
	**Sr [%T·min]**	34.28 ± 4.95	38.28 ± 4.05	35.42 ± 5.12	40.39 ± 4.75
Clot stabilization phase	**Tc [s]**	186.80 ± 18.10	203.40 ± 21.15	**219.80 ± 19.82**	291.20 ± 85.51
	**Sc [%T·min]**	118.15 ± 17.19	147.25 ± 19.89	**150.45 ± 27.59**	199.56 ± 62.95
Fibrinolysis phase	**Lmax [%T]**	39.28 ± 3.44	**44.88 ± 1.44**	42.44 ± 4.87	42.68 ± 7.86
	**Tl [s]**	149.60 ± 17.73	145.20 ± 20.55	142.80 ± 22.48	99.20 ± 69.25
	**Lvo [%T/min]**	22.12 ± 3.56	23.12 ± 1.47	23.16 ± 1.71	19.34 ± 5.49
	**Sf [%T·min]**	49.20 ± 11.51	52.34 ± 9.42	48.99 ± 8.92	63.23 ± 16.63
Overal potential of clot formation and fibrinolysis	**T [s]**	451.65 ± 35.27	458.28 ± 35.84	471.16 ± 37.24	614.14 ± 147.65
	**CL_{AUC} [%T·min]**	201.63 ± 32.08	238.46 ± 30.18	234.34 ± 39.88	303.11 ± 79.77

Tt – thrombin time, Fmax – maximum clotting, Tf – plasma clotting time, Fvo – initial plasma clotting velocity, Sr – area under the clot formation curve; Tc – clot stabilization time, Sc – area under the curve of stable clot formation; Lmax – maximum lysis, Tl – fibrinolysis time, Lvo – initial clot fibrinolysis velocity, Sf – area under the fibrinolysis curve, CL_{AUC} - overall potential of clot formation and fibrinolysis, T - total time of the process of clot formation and fibrinolysis.

Table S6. *The effects of compound* ***10*** *on the kinetic parameters of clot formation and fibrinolysis (CL test). The results are presented as mean ±SD, n = 5 – 6. The values in bold denotes statistically significant difference versus control (p < 0.05).*

CL-test	Parameter	Control	Compound 10		
			**0.1 µmol/mL**	**0.5 µmol/mL**	**1.0 µmol/mL**
Clot formation phase	**Tt [s]**	31.68 ± 5.27	26.53 ± 1.84	33.90 ± 3.72	35.40 ± 6.80
	**Fmax [%T]**	38.20 ± 5.64	40.10 ± 1.84	30.60 ± 6.04	24.77 ± 11.42
	**Tf [s]**	88.43 ± 10.23	**75.02 ± 4.15**	**79.90 ± 8.33**	84.44 ± 10.91
	**Fvo [%T/min]**	57.58 ± 8.27	65.17 ± 9.99	51.70 ± 8.79	49.47 ± 21.76
	**Sr [%T·min]**	39.12 ± 8.25	33.07 ± 2.47	**26.87 ± 4.48**	27.48 ± 3.44
Clot stabilization phase	**Tc [s]**	194.00 ± 47.73	173.00 ± 10.49	226.33 ± 18.74	281.20 ± 48.18
	**Sc [%T·min]**	126.97 ± 37.92	112.30 ± 11.69	112.64 ± 26.32	145.85 ± 42.98
Fibrinolysis phase	**Lmax [%T]**	40.32 ± 4.01	41.80 ± 1.62	34.02 ± 8.24	**35.65 ± 1.74**
	**Tl [s]**	158.67 ± 38.50	143.00 ± 4.60	**210.17 ± 30.52**	**246.50 ± 55.79**
	**Lvo [%T/min]**	23.77 ± 5.72	19.80 ± 2.77	**14.74 ± 2.56**	**12.74 ± 4.33**
	**Sf [%T·min]**	58.61 ± 20.55	45.44 ± 3.80	43.15 ± 5.27	44.07 ± 8.61
Overal potential of clot formation and fibrinolysis	**T [s]**	472.78 ± 92.75	417.55 ± 9.78	**550.30 ± 43.20**	647.84 ± 103.30
	**CL_{AUC} [%T·min]**	224.74 ± 66.16	190.82 ± 16.30	182.64 ± 34.90	227.84 ± 70.43

Tt – thrombin time, Fmax – maximum clotting, Tf – plasma clotting time, Fvo – initial plasma clotting velocity, Sr – area under the clot formation curve; Tc – clot stabilization time, Sc – area under the curve of stable clot formation; Lmax – maximum lysis, Tl – fibrinolysis time, Lvo – initial clot fibrinolysis velocity, Sf – area under the fibrinolysis curve, CL_{AUC} - overall potential of clot formation and fibrinolysis, T - total time of the process of clot formation and fibrinolysis.
